# Enhanced Single‐Particle Upconversion Imaging via Energy Migration Boosting

**DOI:** 10.1002/advs.202510624

**Published:** 2025-08-27

**Authors:** Yanxin Zhang, Rongrong Wen, Tianli Zhai, Wenrui Zhang, Fan Ding, Huan Ling, Yunxiang Zhang, Qian Liu

**Affiliations:** ^1^ Department of Chemistry and Shanghai Key Laboratory of Molecular Catalysis and Innovative Materials Fudan University Shanghai 200438 China

**Keywords:** axonal transport, energy migration, single‐particle imaging, upconversion luminescence

## Abstract

Lanthanide‐doped upconversion nanoparticles (UCNPs) are promising bioimaging probes due to their exceptional photostability and minimal background interference. However, their limited single‐particle brightness has hindered broader applications. The study addresses this challenge by enhancing energy migration (EM) between sensitizer Yb^3+^ to improve energy transfer efficiency to emitter Er^3+^. Nanoparticles are designed with a sensitizer/emitter‐segregated core‐shell‐shell architecture (NaLu_0.9_Er_0.1_F_4_@NaYbF_4_@NaLuF_4_) to inhibit back energy transfer (BET) and then increased Yb^3+^ doping levels (NaLu_0.9‐x_Yb_x_Er_0.1_F_4_@NaYbF_4_@NaLuF_4_) to enhance EM into the core. UCNPs with an alloy‐core of NaYb_0.9_Er_0.1_F_4_ exhibited the brightest upconversion luminescence, achieving over a tenfold enhancement compared to NaLu_0.9_Er_0.1_F_4_‐core counterparts, highlighting the importance of EM. Further optimization of the Yb^3+^/Er^3+^ ratio and inert shell thickness (NaLuF_4_) maximized single‐particle brightness. These optimized UCNPs enabled long‐term tracking of axonal transport in live dorsal root ganglion (DRG) neurons. Using a Bayesian Hidden Markov Model, it quantitatively characterized resolved heterogeneous motion states and annotated trajectories with local spatiotemporal dynamics of retrograde, anterograde, and diffusive motions. The analysis revealed a kinesin‐dynein coordination mechanism, where anterograde motion facilitates retrograde activation. It also examined the effects of inhibitors and stimulants on transport behavior. These findings establish upconversion single‐particle tracking (uSPT) as a powerful tool for long‐term, real‐time monitoring of neuronal activities.

## Graphical abstract

A core‐shell‐shell Yb^3^⁺/Er^3^⁺ UCNP architecture is engineered to balance energy migration and back energy transfer, yielding more than a tenfold enhancement in single‐particle brightness. Leveraging these optimized probes, it achieves long‐term, high‐resolution single‐particle tracking in live neurons, uncovering coordinated transport mechanisms of motor proteins.

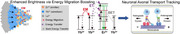

## 1 Introduction

Cargo transport along axons in neurons is a crucial physiological process that ensures proper neuronal function and maintenance.^[^ ^1^, ^2^, ^3^ ^]^ Axons, the long, slender projections of neurons, facilitate the transport of essential materials, such as proteins, organelles, and signaling molecules between the cell body and synaptic terminals.^[^ ^4^, ^5^ ^]^ This transport is bidirectional: anterograde transport moves materials from the cell body to the synapse, while retrograde transport carries materials back to the cell body for recycling or signaling.^[^ ^6^, ^7^, ^8^ ^]^ The microtubule network within axons serves as tracks for motor proteins like kinesin and dynein, which actively shuttle cargo to its destination.^[^ ^9^, ^10^, ^11^ ^]^ Proper cargo transport is vital for neuronal health and function, as disruptions can lead to neurodegenerative diseases and impaired neural communication.^[^ ^12^, ^13^ ^]^

Axonal transport is a relatively lengthy process, and current single‐particle tracking (SPT) techniques have limited tracking duration, presenting a major challenge in this field of research. SPT using upconversion nanoparticles (UCNPs) with their excellent photostability and resistance to photodegradation, has greatly enhanced our ability to visualize and study axonal transport in live neurons, offering deeper insights into its underlying mechanisms.^[^ ^7^, ^14^, ^15^, ^16^, ^17^, ^18^, ^19^, ^20^ ^]^ However, a notable limitation of UCNPs is their relatively low brightness at the single‐particle level.^[^ ^21^, ^22^, ^23^, ^24^, ^25^, ^26^ ^]^

**Figure 1:**
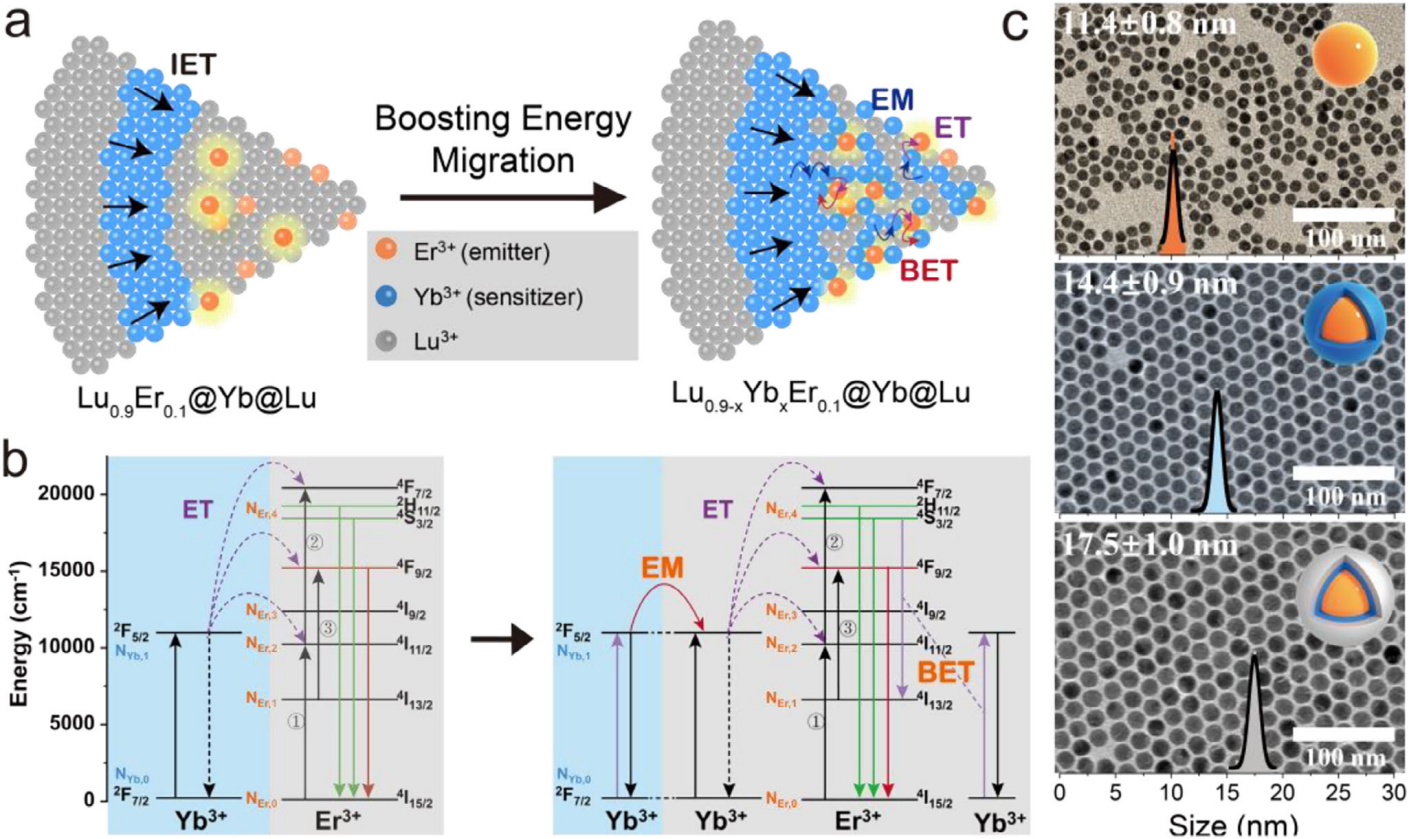
Synthesis and characterization of Lu_0.9‐x_Yb_x_Er_0.1_@Yb@Lu UCNPs. a) Schematic illustrating the design strategy: incorporation of Yb^3+^ sensitizer ions into the core of Lu_0.9‐x_Yb_x_Er_0.1_@Yb@Lu UCNPs. b) Energy transfer mechanism: schematics depicting energy flow leading to upconversion luminescence. EM: Energy migration, IET: interfacial energy transfer, BET: back energy transfer. c) Representative TEM images of Yb_0.9_Er_0.1_@Yb@Lu UCNPs, showing from top to bottom the core, the core‐shell and the core‐shell‐shell architectures. Insets: corresponding size distribution histograms with Gaussian fits.

The weak emission of UCNPs can be attributed to several intrinsic factors: i) low absorption cross‐section^[^ ^27^, ^28^, ^29^ ^]^; ii) non‐radiative relaxation, such as phonon interactions within the host matrix and cross‐relaxation processes;^[^ ^30^, ^31^, ^32^, ^33^, ^34^ ^]^ iii) back energy transfer (BET) from emitter ions to sensitizer ions or to other quenching sites, decreasing upconversion efficiency.^[^ ^35^, ^36^, ^37^ ^]^ Efforts to enhance the luminescence of lanthanide‐based UCNPs often focus on addressing these challenges. To address BET, researchers have developed a strategy to spatially separate the sensitizer and emitter, thereby inhibiting BET and improving the brightness of upconversion luminescence.^[^ ^38^, ^39^, ^40^, ^41^, ^42^ ^]^ We have conducted a study on the topologically segregated core‐shell architecture and demonstrated that an outside‐in energy transfer pattern, where emitters are confined to the core and sensitizers are placed in the interior shell, can significantly enhance upconversion luminescence (UCL), highlighting the efficiency of interfacial energy transfer (IET, **Figure** **1**a).^[^ ^39^ ^]^ However, this topological segregation of sensitizers and emitters can also hinder the energy migration (EM) process between Yb^3+^, potentially limiting the activation of emitter ions located deeper within the core center.

To explore the importance of EM in UCL, we introduced sensitizers in the core, adjusting their concentration (NaLu_0.9‐x_Yb_x_Er_0.1_F_4_@NaYbF_4_@NaLuF_4_), and studied their single particle luminescence performance (Figure 1a,b). Precision engineering of nanostructures to enhance EM and inhibit BET, coupled with meticulous control of the inert shell, played a crucial role in achieving improved energy transfer and optimizing particle brightness. We utilized the optimal UCNPs for long‐term tracking in live neurons. This provided valuable insights into axonal transport processes, validating cargo transport models within neurons.

## 2 Results and Discussion

To gain insights into the energy transfer process, we conducted theoretical analysis, focusing on five processes:^[^ ^31^, ^43^, ^44^ ^]^ three energy transfer upconversion (ETU) events from Yb^3+^ to Er^3+^, cross‐relaxation (CR) between Er^3+^ ions, and BET from Er^3+^ to Yb^3+^. (details in Figure S1, Supporting Information). With an increase in Yb^3+^ content within the core, EM was enhanced. Both BET (N_Yb,1_ + N_Er,4_ → N_Yb,0_ + N_Er,1_) and ETU‐3 (N_Yb,0_ + N_Er,1_ → N_Yb,1_ + N_Er,3_) effects also exhibited an upward trend, amplifying the population of ^4^F_9/2_ (Er^3+^) while decreasing the populations of ^4^H_11/2_/^4^S_3/2_ (Er^3+^) state. This shift could result in an increased red‐to‐green (R/G) emission ratio. Importantly, as the concentration of core sensitizers increases, the positive impact of improved EM outweighs the negative effects of BET, ultimately leading to enhanced UCL.

To verify the theoretical analysis and enhance the single particle brightness, we designed and synthesized a series of core‐shell‐shell UCNPs (NaLu_0.9‐x_Yb_x_Er_0.1_F_4_@NaYbF_4_@NaLuF_4_, referred to as Lu_0.9‐x_Yb_x_Er_0.1_@Yb@Lu) with varying doping concentrations of Yb^3+^ ions within the core, ranging from 0 to 90%. These UCNPs were synthesized using a solvothermal method to achieve precise control over nanoparticle formation. The resulting cores were ≈11 nm in size. An interior shell of NaYbF_4_ was epitaxially grown on the cores, resulting in core‐shell particles with a size of ≈14 nm. Additionally, a 1.5 nm inert shell was applied to prevent surface quenching. The size and morphology of these nanoparticles were characterized using transmission electron microscopy (TEM; Figure 1c; Figure S2, Supporting Information), which revealed uniform size and monodispersity across all samples. High‐resolution transmission electron microscopy (HR‐TEM) and X‐ray diffraction (XRD) measurements (Figure S3, Supporting Information) confirmed that these UCNPs possess a hexagonal phase. Even at the highest Yb^3+^ doping level (90%), the diffraction peak positions remained consistent with those of the standard β‐NaREF_4_ phase, while the measured lattice spacings from HR‐TEM matched well with the planes. Minor peak broadening was observed, which may be attributed to local lattice strain induced by heavy Yb^3+^ substitution,^[^ ^45^ ^]^ but the overall crystallinity and phase purity remained high, supporting efficient energy migration.

We then explored the ensemble upconversion luminescence properties of these UCNPs. As shown in Figure S4a,b (Supporting Information), the UCL spectra revealed significant emissions at 521, 541, and 654 nm, corresponding to the radiative transitions from Er^3+ 4^I_11/2_, ^4^S_3/2_, and ^4^F_9/2_ states to Er^3+ 4^I_15/2_ state, respectively. The UCL intensity gradually increased with higher Yb^3+^ doping concentration, consistent with our theoretical analysis. To gain deeper insight into the mechanism driving this enhancement, we studied the power dependence of UCL for these UCNPs (Figure S4c,d, Supporting Information). As shown in Figure S4e (Supporting Information), the power‐dependent slope decreased progressively with increasing Yb^3+^ doping, suggesting that higher Yb^3+^ doping in the core leads to a faster saturation of the upconversion luminescence due to increased energy absorption and migration. Additionally, we observed that the slope of the red emission was steeper than that of the green emission, indicating a stronger power dependence of the red emission.

**Figure 2:**
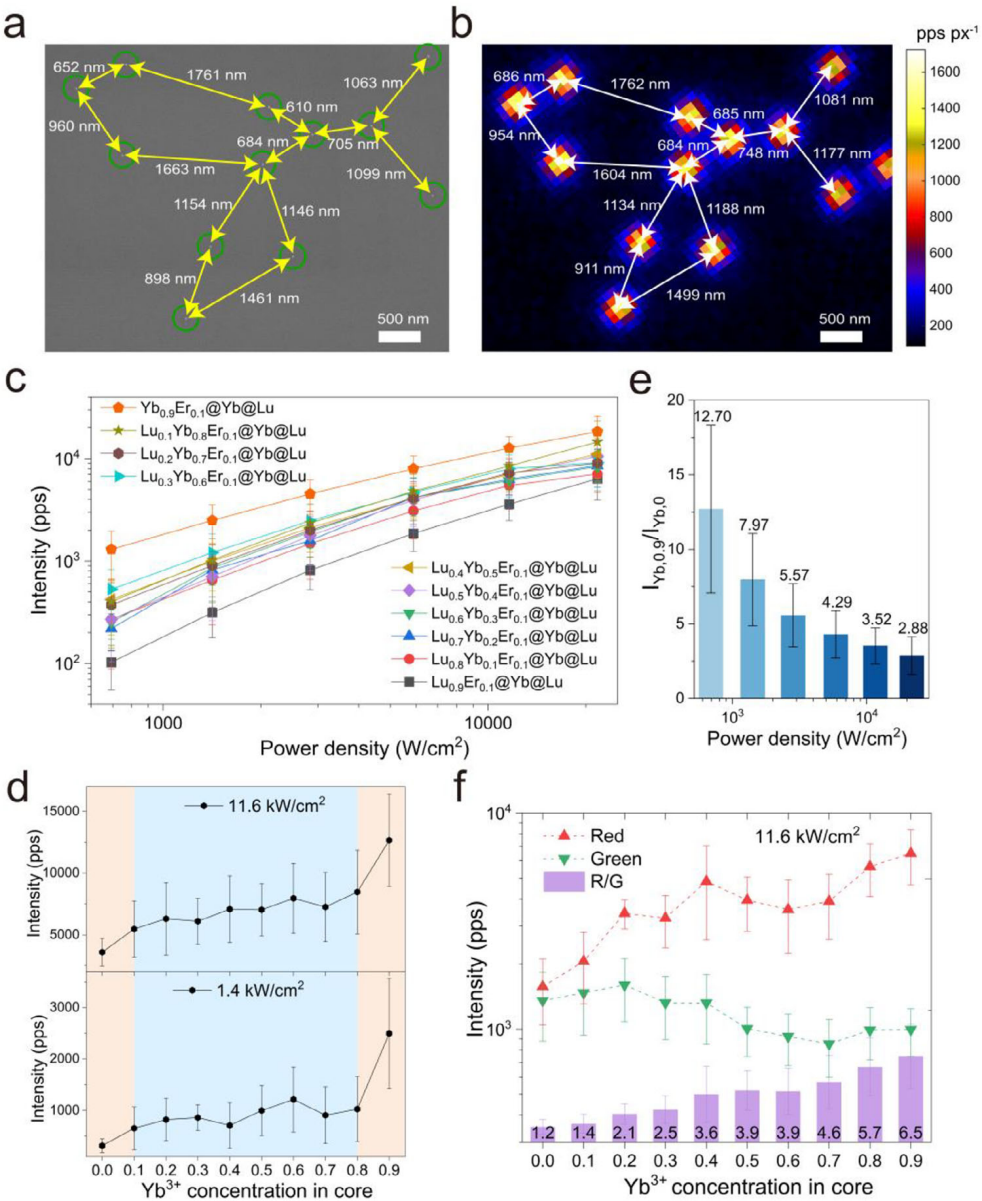
Single‐Particle characterizations of Lu_0.9‐x_Yb_x_Er_0.1_@Yb@Lu UCNPs. a) Wide‐field luminescence image of Yb_0.9_Er_0.1_@Yb@Lu at 21.7 kW cm^−2^. Scale bar, 500 nm. b) SEM image corresponding to the corresponding region in (a). c) Single‐particle upconversion luminescence intensity as a function of power density (0.69–21.7 kW cm^−2^), acquired with wide‐field microscopy using a 750 nm short‐pass filter. d) Single‐particle upconversion luminescence intensity of Lu_0.9‐x_Yb_x_Er_0.1_@Yb@Lu at 11.6 kW cm^−2^ and 1.4 kW cm^−2^. e) Brightness ratios of Yb_0.9_Er_0.1_@Yb@Lu UCNPs (the brightest) and Lu_0.9_Er_0.1_@Yb@Lu UCNPs (the darkest). f) Green and red emission intensities and corresponding R/G ratios of single UCNPs at 11.6 kW cm^−2^, acquired using ET535/70m (green) and ET645/75m (red) filters. All single‐particle statistics results were presented as means ± standard deviation (three independent experiments, more than five field of views of wide‐field images were acquired for each experiments; "n" represents the number of nanoparticles, Lu_0.9_Er_0.1_@Yb@Lu: n = 313, Lu_0.8_Yb_0.1_Er_0.1_@Yb@Lu: n = 527, Lu_0.7_Yb_0.2_Er_0.1_@Yb@Lu: n = 415, Lu_0.6_Yb_0.3_Er_0.1_@Yb@Lu: n = 226, Lu_0.5_Yb_0.4_Er_0.1_@Yb@Lu: n = 355, Lu_0.4_Yb_0.5_Er_0.1_@Yb@Lu: n = 238, Lu_0.3_Yb_0.6_Er_0.1_@Yb@Lu: n = 713, Lu_0.2_Yb_0.7_Er_0.1_@Yb@Lu: n = 625, Lu_0.1_Yb_0.8_Er_0.1_@Yb@Lu: n = 426, Yb_0.9_Er_0.1_@Yb@Lu: n = 686), "pps" means photons per second, and "pps px^−1^″ means photons per second per pixel.

To evaluate the performance of these UCNPs under higher irradiance, we conducted single‐particle characterizations. Alignment of single‐particle UCL images with corresponding scanning electron microscope (SEM) images revealed a clear spatial correspondence among individual particles, confirming that the UCL signal originated from distinct single particles (**Figure** **2**a,b). We quantified the intensity of each particle under continuous wave wide‐field illumination at 980 nm, using excitation power densities ranging from 0.69 to 21.7 kW cm^−2^ (Figure 2c). Consistent with our ensemble results, single‐particle brightness increased with higher sensitizer concentrations in the core. However, while incorporating sensitizer ions into the core improves the light harvesting ability and facilitates EM within the core, it also leads to an increase in BET. These positive and negative effects compete, leading to non‐monotonic UCL behavior rather than a straightforward enhancement. At medium Yb^3+^ doping concentration, a distinct plateau in the UCL intensity was observed (Figure 2d; Figure S5, Supporting Information), indicating a balance between positive EM and negative BET effects. Increasing the Yb^3+^ doping from 0 to 10% significantly enhanced EM, while the negative impact of BET remained minimal, resulting in a notable brightness enhancement. At the highest doping concentration with 90% Yb^3+^, BET became saturated as sufficient Yb^3+^ ions surrounded each Er^3+^ ion, enhanced EM and light harvest ability boosted the UCL emission. As a result, the single‐particle upconversion brightness as a function of Yb^3+^ doping concentration in the core followed an S‐shaped curve.

Moreover, we quantified enhancement factor by comparing the brightness of Yb_0.9_Er_0.1_@Yb@Lu and Lu_0.9_Er_0.1_@Yb@Lu (Figure 2e), which showed a clear power dependence. At a power density of 0.69 kW cm^−2^, the enhancement factor increased by over an order of magnitude. In contrast, at 21.7 kW cm^−2^, the brightness of Yb_0.9_Er_0.1_@Yb@Lu only showed a 2.88‐fold increase compared to Lu_0.9_Er_0.1_@Yb@Lu. The enhancement factor under stronger irradiation is consistent with the improved light harvest capacity in Yb_0.9_Er_0.1_@Yb@Lu, stemming from its 1.9‐fold increased Yb^3+^ content compared to Lu_0.9_Er_0.1_@Yb@Lu. This correlation further suggests that the pronounced enhancement observed under lower irradiance primarily arises from the formation of a highly connected Yb^3+^ energy‐migration network in Yb_0.9_Er_0.1_@Yb@Lu, which enables more efficient energy transfer to Er^3+^ activators.

We further performed multicolor single‐particle imaging using band‐pass filters to separately collect green and red emissions (Figure 2f; Figure S6, Supporting Information). The red emission appeared stronger than the green emission and displayed an enhancement as the Yb^3+^ concentration increased, whereas the green emission showed an opposite trend. This phenomenon can be attributed to the BET process, which increased the population of red‐emitting energy levels through relaxation from the green‐emitting energy levels, resulting in a dynamic where red emission intensity rises while the green emission intensity declines.^[^ ^37^, ^46^, ^47^, ^48^ ^]^ The single‐particle R/G ratio consistently increased with higher Yb^3+^ concentrations, consistent with our ensemble measurements (Figure S4f, Supporting Information). We also observed that the R/G ratio increased with power density and exhibited a similar power dependence across different Yb^3+^ ion concentrations. In brief, incorporating sensitizer ions into the core boosts the red emission brightness of UCNPs, with an “alloy‐core” structure consisting solely of sensitizer and emitter ions, yielding optimal luminescence performance.^[^ ^49^, ^50^ ^]^

**Figure 3:**
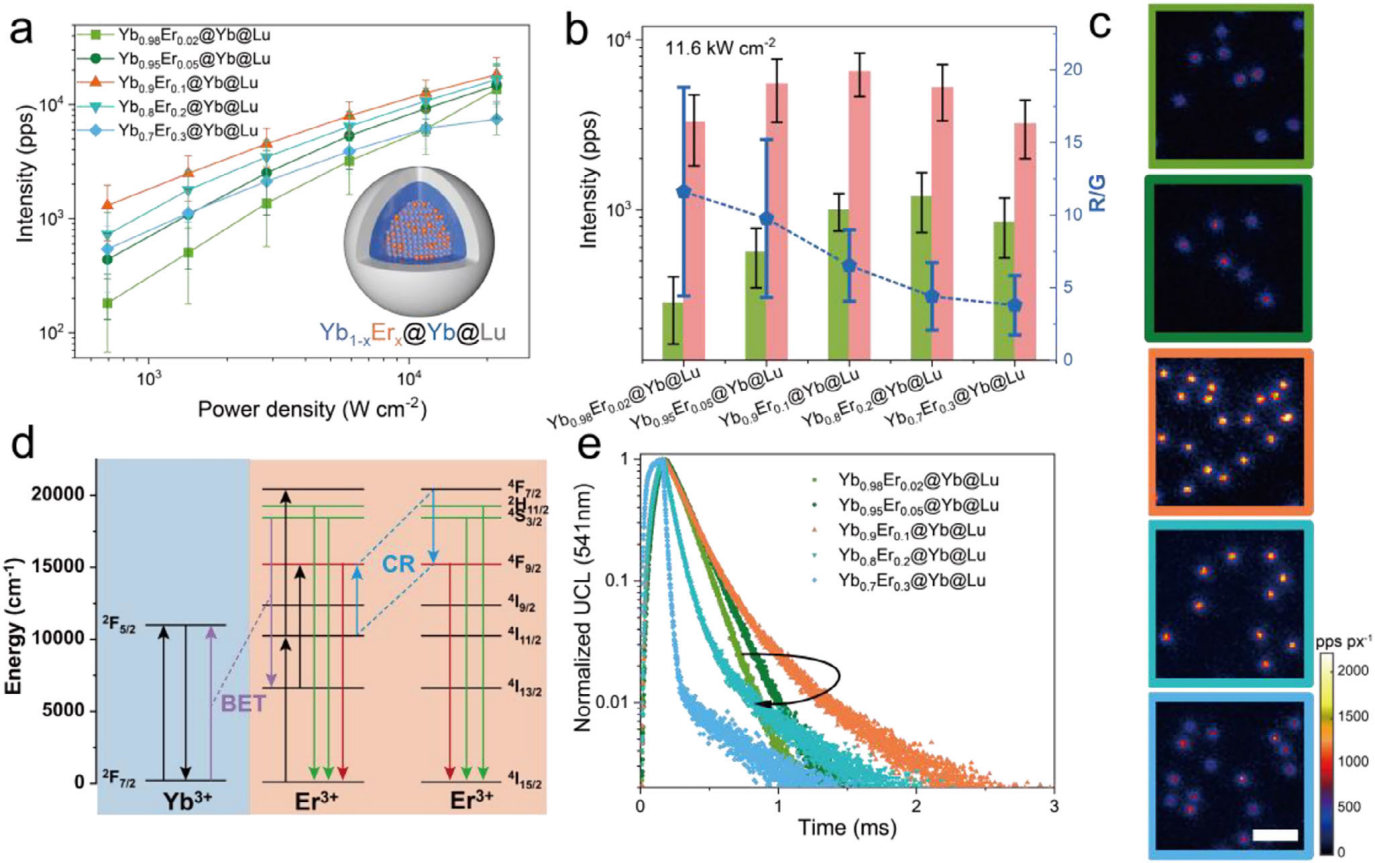
Optimizing sensitizer‐emitter ratio in the core of Yb_1‐x_Er_x_@Yb@Lu UCNPs. a) Single‐particle upconversion luminescence intensity as a function of power density (0.69–21.7 kW cm^−2^), acquired with wide‐field microscopy using a 750 nm short‐pass filter. b) Single‐particle red and green emission intensities and their corresponding R/G ratios at 11.6 kW cm^−2^ under 980 nm laser irradiation. All single‐particle statistics results were presented as means ± standard deviation (three independent experiments, more than five field of views wide‐field images were acquired for each experiment; Yb_0.98_Er_0.02_@Yb@Lu:n = 615, Yb_0.95_Er_0.05_@Yb@Lu:n = 508, Yb_0.9_Er_0.1_@Yb@Lu:n = 958, Yb_0.8_Er_0.2_@Yb@Lu:n = 381, Yb_0.7_Er_0.3_@Yb@Lu:n = 270). c) Wide‐field single‐particle luminescence images acquired using a 750 short‐pass filter at 21.7 kW cm^−2^ under 980 nm laser irradiation. d) Schematic illustrating the energy transfer process, incorporating cross‐relaxation (CR) and BET. e) Luminescence decay curves at 541 nm emission, excited by a 980 nm pulsed laser.

We further optimized the ratio of sensitizer to emitter in the “alloy‐core” architecture. The emitter (Er^3+^) doping concentration was varied from 2% to 30%, while the sensitizer (Yb^3+^) concentration was adjusted from 98% to 70%. Comprehensive morphological characterization and ensemble properties measurements were conducted, as depicted in Figures S7 and S8 (Supporting Information). Single‐particle saturation curves were obtained for all samples, revealing that the Yb_0.9_Er_0.1_@Yb@Lu UCNPs exhibited the highest single‐particle emission across all tested irradiances (**Figure** **3**a,c). Single‐particle brightness increased with higher Er^3+^ concentrations in the “alloy‐core” structure, peaking at 10%, after which it gradually declined. A decreasing trend in the R/G ratio was observed as the concentration of Er^3+^ ions increased (Figure 3b; Figure S9, Supporting Information). To better understand this phenomenon, we analyzed the energy transfer process (Figure 3d).^[^ ^23^, ^35^, ^51^ ^]^ The observed R/G emission ratio trend suggests a reduced contribution from BET, which preferentially quenches green emission over red.^[^ ^52^, ^53^ ^]^ Concurrently, the decrease in overall brightness at higher Er^3+^ contents can be attributed to the enhanced CR, which introduced nonradiative loss pathways, especially at elevated doping levels.^[^ ^23^, ^54^, ^55^ ^]^ This interpretation is supported by the lifetimes of both green and red emissions, which reached their maxima at 10% Er^3+^ doping—corresponding to the highest brightness observed. Together, these findings indicate that while reduced BET yields a more balanced R/G emission ratio, CR becomes the predominant loss mechanism at higher Er^3^⁺ concentrations, leading to substantial brightness reduction and constraining practical performance.

**Figure 4:**
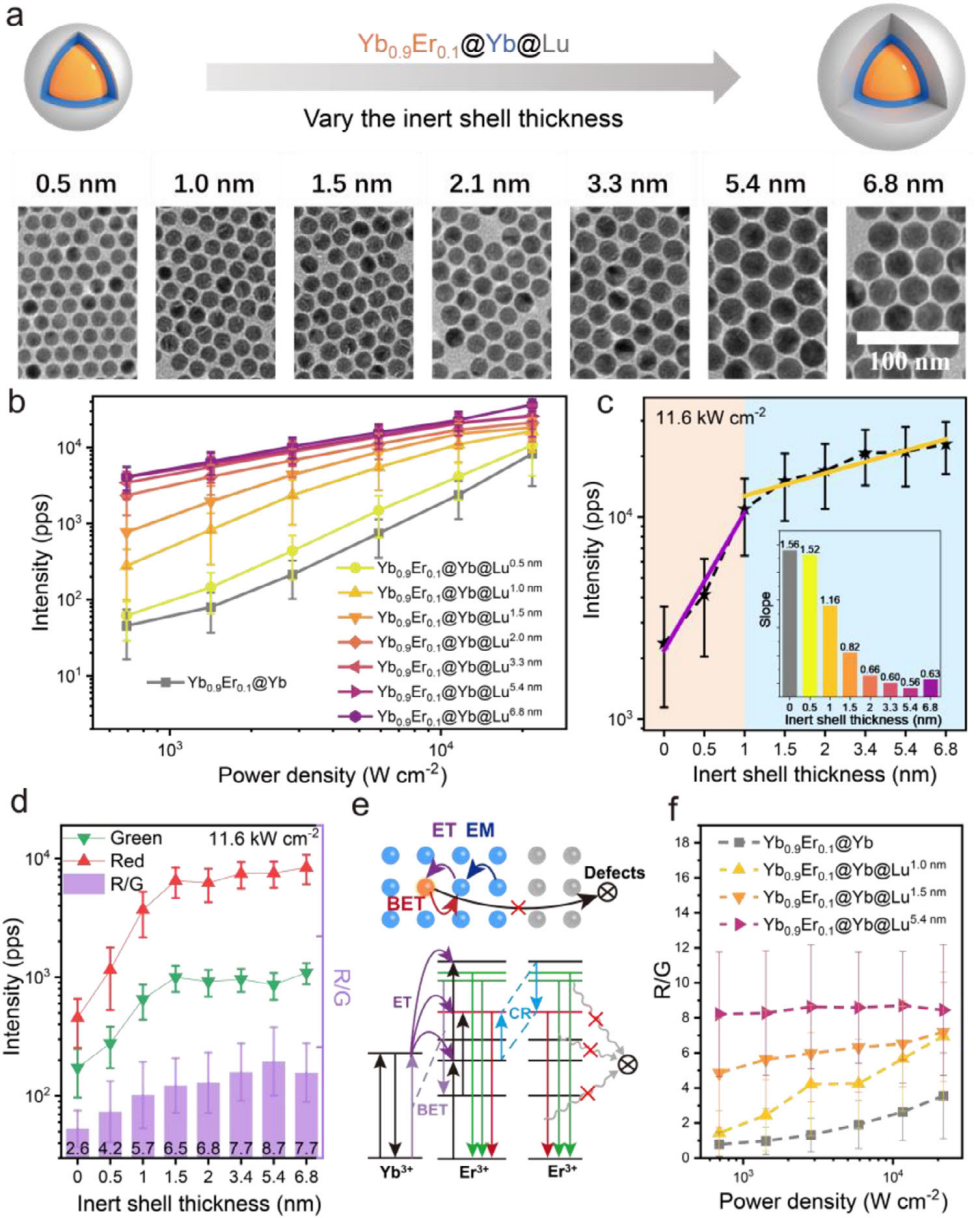
Influence of inert shell thickness on Yb_0.9_Er_0.1_@Yb@Lu UCNPs single‐particle brightness. a) Schematic illustrating the designs with varying inert shell thicknesses of Yb_0.9_Er_0.1_@Yb@Lu UCNPs, alongside representative TEM images showcasing the resulting morphology. b) Single‐particle upconversion luminescence intensity as a function of power density (0.69–21.7 kW cm^−2^), acquired with wide‐field microscopy using a 750 nm short‐pass filter. c) Single‐particle upconversion luminescence intensity of Yb_0.9_Er_0.1_@Yb@Lu with different inert shell thickness at 11.6 kW cm^−2^. Inset: Slope of single‐particle upconversion luminescence intensity respect to excitation power density. d) Single‐particle red and green emission intensities and the corresponding R/G ratio at 11.6 kW cm^−2^. e) Schematic representations depicting the influence of an inert shell on energy transfer. ET: energy transfer, EM: energy migration, BET: back energy transfer. f) The R/G ratio of Yb_0.9_Er_0.1_@Yb, Yb_0.9_Er_0.1_@Yb@Lu^1.0 nm^, Yb_0.9_Er_0.1_@Yb@Lu^1.5 nm^ and Yb_0.9_Er_0.1_@Yb@Lu^5.4 nm^ at various power densities. All single‐particle statistics results were presented as means ± standard deviation (three independent experiments, more than five field of views wide‐field images were acquired for each experiment; 0 nm:n = 459, 0.5 nm:n = 258, 1.0 nm:n = 260, 1.5 nm:n = 460, 2.0 nm:n = 550, 3.3 nm:n = 656, 5.4 nm:n = 523, 6.8 nm:n = 213).

Based on the previously optimized Yb_0.9_Er_0.1_ “alloy‐core” architecture, we explored the influence of inert shell thickness on single‐particle performance. We varied the thickness of the inert shell ranging from 0.5 to 6.8 nm (**Figure** **4**a; Figure S10, Supporting Information). At the single‐particle level, all samples displayed a consistent trend of power‐dependent behavior (Figure 4b; Figure S11, Supporting Information). Compared to UCNPs without an inert shell, brightness increased significantly with the addition of an inert shell. At 11.6 kW cm^−^^2^, a notable brightness enhancement was observed even with a 0.5 nm inert shell (Figure 4c). As the shell thickness increased to ≈1 nm, linear relationship between shell thickness and brightness enhancement emerged. However, beyond this point, further increases in thickness led to a gradual reduction in enhancement, with brightness eventually plateauing. To better understand the correlation between inert shell thickness and upconversion luminescence, we conducted a comparative analysis for both red and green emissions at the single‐particle level and analyzed their R/G ratio (Figure 4d; Figure S12, Supporting Information). Both red and green emissions, as well as total brightness, exhibited a similar enhancement pattern: a rapid initial increase in intensity followed by a slower rise as shell thickness increased. The R/G ratio showed a similar trend with the brightness. When the active core was adequately shielded, the energy transfer process stabilized, leading to a consistent R/G ratio (Figure 4e). In contrast, insufficient protection caused the R/G ratio to increase with power density. Once the active component is properly shielded, the energy transfer process remains unaffected by external environmental factors, thus the R/G ratio remains predominantly stable regardless of power levels (Figure 4f). Balancing luminance enhancement and nanoparticle size, we selected a 1.5 nm inert shell to protect the active component, ensuring optimal performance. This thickness was selected based on single‐particle brightness measurements, which identified it as the optimal configuration for live‐cell imaging applications among sub‐20 nm particles.^[^ ^56^ ^]^ Moreover, maintaining the overall hydrodynamic size within this range benefits intracellular diffusivity and enhanced endocytic uptakes in neuronal cells, as supported by previous studies.^[^ ^57^, ^58^ ^]^

**Figure 5:**
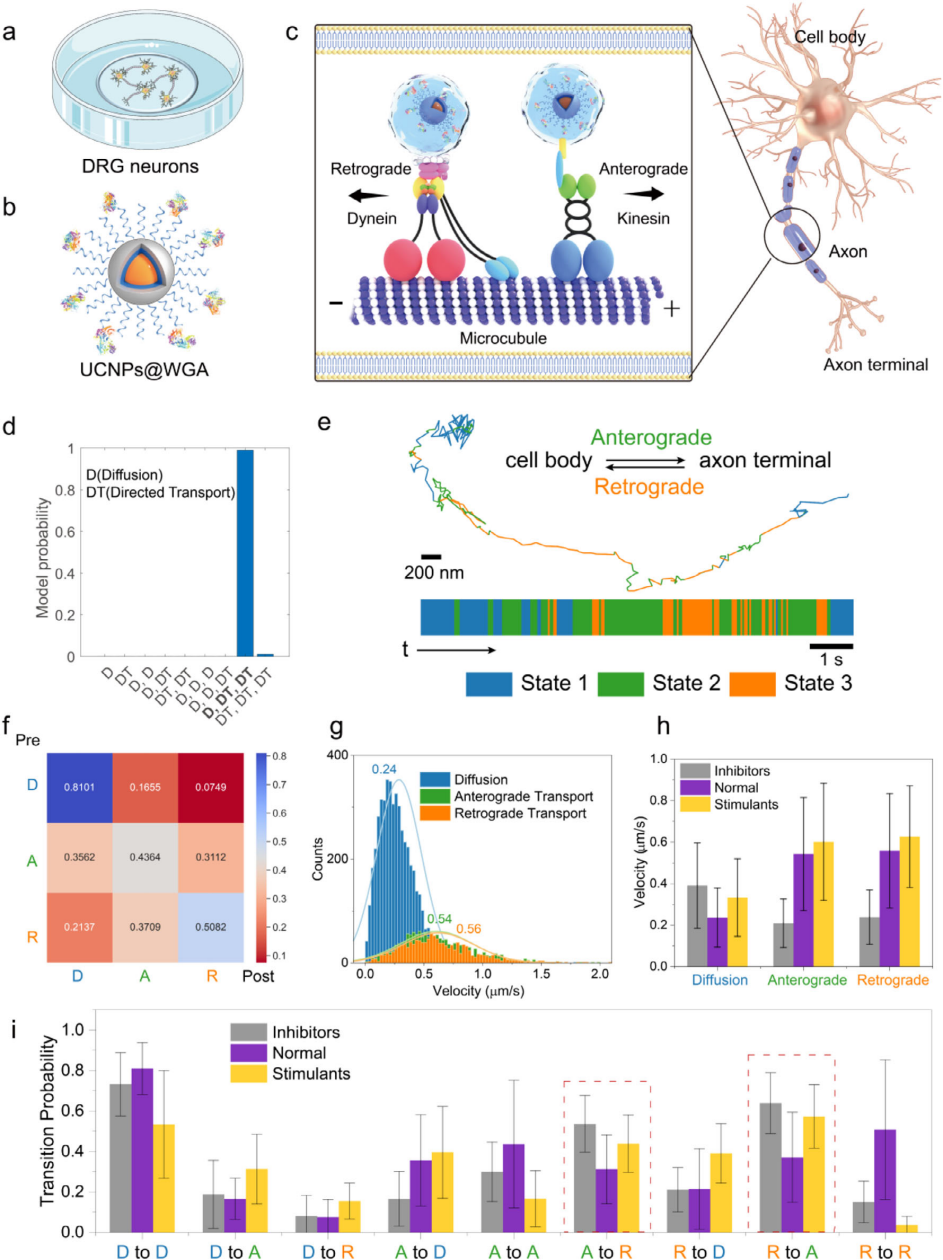
Upconversion Single‐Particle Tracking of Axonal Transport in DRG Neurons. a) Schematic illustrating dorsal root ganglion (DRG) neuronal growth in a glass‐bottom Petri dish. b) Schematic of UCNPs@WGA complex. c) Illustrations depicting anterograde and retrograde transport processes within neurons using UCNPs@WGA. d) Model probabilities for all motion models evaluated in the HMM‐Bayes analysis. e) Representative axonal transport trajectory and corresponding HMM state assignment. f) Transition probabilities and g) Velocities distributions for the three HMM‐Bayes states derived from all axonal transport trajectories. h) Impact of stimulants dopamine (DA, 30 µM for 2 h) and inhibitors ciliobrevin D (cD, 30 µM for 2 h) on velocities distributions and i) transition probabilities of the three states. The results of h‐i were presented as means ± standard deviation (two independent experiments, more than twenty trajectories were acquired for each experiment).

We utilized optimized UCNPs (≈17 nm Yb_0.9_Er_0.1_@Yb@Lu) as probes for tracking within neurons. Neurons consist of cell bodies, dendrites, and axons, with cargo transport along axons being a critical process for neuronal function. This transport facilitates long‐distance material exchange between cell bodies and synapses. Kinesin promotes anterograde transport along microtubules to the synapse, while dynein facilitates retrograde transport from the synapse back to the cell body (**Figure** **5**c). For high spatiotemporal resolution and long‐term monitoring of axonal transport in live neurons, photostable and background‐free UCNPs are ideal probes. Unlike methods that require microfluidic devices to constrain neuronal growth, we cultured neurons directly on a glass substrate, providing ample free space for neuronal growth (Figure 5a) to mimic natural neuronal growth conditions.

To ensure uniform dispersion in aqueous media, the UCNPs were functionalized with DSPE‐mPEG, a modification well known to improve colloidal stability, reduce nonspecific interactions, and prolong blood circulation in vivo by minimizing protein adsorption and reticuloendothelial system clearance.^[^ ^59^, ^60^, ^61^ ^]^ The resulting PEGylated UCNPs exhibited stable emission intensity across a broad pH range (pH 2.09–9.94, 37 °C; Figure S13, Supporting Information) and maintained their structural integrity after 24 h incubation in PBS, with or without 10% fetal bovine serum (FBS; Figure S14, Supporting Information). Cytocompatibility assays with neurons showed >80% cell viability at a concentration up to 200 nmol mL^−1^ after 24 h exposure (Figure S15, Supporting Information), confirming their suitability for live‐cell imaging. The UCNPs were coated with a mixture of DSPE‐mPEG (M.W. 2000) and DSPE‐mPEG‐NHS (w/w, 5:1). The NHS group was then conjugated with the NH_2_ moiety on wheat germ agglutinin (WGA) protein surfaces, a lectin that specifically binds glycoproteins on cell surfaces,^[^ ^62^, ^63^, ^64^ ^]^ forming UCNPs@WGA complexes (Figure 5b). To simulate probe capture and transport within a growing neuronal network, UCNPs@WGA complexes were incubated in a DRG culture dish. Their dynamics were monitored under 980 nm laser excitation, enabling single‐particle tracking.

To resolve the complexity of axonal transport dynamics, we employed the Bayesian Hidden Markov Model (HMM‐Bayes) framework to analyze individual particle trajectories.^[^ ^65^, ^66^ ^]^ This approach models distinct motion states along trajectories, using Bayesian model selection to identify the simplest stochastic model fitting observed displacements. Model probabilities for evaluated motion are shown in Figure 5d. The three‐state model “D, DT, DT” exhibited the highest probability, indicating one diffusive state and two active transport states‐retrograde and anterograde transport. A representative example of axonal transport trajectories is shown in Figure 5e, with transport directionality determined by precise localization of the cell body and axon terminal. Using HMM‐Bayes, trajectories were adaptively classified on a temporal scale, providing detailed annotations of spatiotemporal dynamics, including the timing and location of retrograde, anterograde, or diffusive motion.

We analyzed the transition probabilities among states (Figure 5f). While each movement mode exhibits a strong tendency to persist in its original state, significant transitions between states. Statistical analyses revealed a rare transition from diffusion state to retrograde transport, suggesting anterograde transport acts as an intermediary between these states. This aligns with the coordinated action of kinesin and dynein.^[^ ^67^, ^68^ ^]^ Kinesin, responsible for anterograde transport, activates dynein for retrograde transport.^[^ ^69^, ^70^ ^]^ For instance, in fruit fly neurons, efficient dynein‐mediated retrograde transport depends on prior kinesin‐driven anterograde transport.^[^ ^69^ ^]^ Beyond cargo delivery to the axon terminal, kinesin may activate dynein via microtubule stabilization or essential signaling cues.^[^ ^68^, ^71^ ^]^ Notably, we present evidence for kinesin‐mediated dynein activation for retrograde transport in DRG neurons, a previously unobserved phenomenon. Furthermore, analogous experiments performed in primary hippocampal neurons showed a similar state transition patterns (Figure S18, Supporting Information), suggesting that this coordinated transport mechanism is also applicable to other types of neurons.

Average velocities for all trajectories across states were quantified (Figure 5g). The diffusive state showed a velocity of 0.24 ± 0.14 µm s^−1^, while anterograde transport (0.54 ± 0.27 µm s^−1^) and retrograde transport (0.56 ± 0.28 µm s^−1^) exhibited comparable average velocities. This suggests that the active transport modes operate at comparable velocities, whereas the diffusive state exhibits slower movement due to its passive, non‐motor‐driven nature.

We further investigated the impact of inhibitors and stimulants on axonal transport using the HMM‐Bayes framework (Figure 5h,i). DRG neurons were pretreated with ciliobrevin D (30 µM, 2 h) or dopamine (30 µM, 2 h) prior to imaging, following established protocols. Inhibitors significantly reduced the velocities of directional transport to below diffusion levels while decreasing state persistence and increasing anterograde‐retrograde transitions. These effects likely reflects disrupted kinesin‐dynein coordination, favoring competitive cargo binding or microtubule tug‐of‐war dynamics, leading to inefficient transport and cargo misrouting.^[^ ^72^, ^73^, ^74^ ^]^ In contrast, stimulants increased velocities across all movement states and enhanced the probability of transitions between anterograde and retrograde transport, likely by modulating motor protein binding dynamics to microtubules and promoting cooperative transport.^[^ ^75^, ^76^, ^77^ ^]^ These results underscore upconversion single‐particle tracking as a powerful tool for high‐resolution, real‐time analysis of axonal transport.

## 3 Conclusion

In this study, we systematically optimized the design of UCNPs to enhance their single‐particle brightness and demonstrated their application in high‐resolution, long‐term tracking of axonal transport in live neurons. By engineering a core‐shell‐shell architecture (Lu_0.9‐x_Yb_x_Er_0.1_@Yb@Lu) with varying Yb^3+^ doping concentrations, we achieved significant improvements in single‐particle luminescence. The incorporation of Yb^3+^ in the core enhanced energy migration, resulting in over an order of magnitude enhancement in luminescence. The optimal “alloy‐core” structure, with a 90% Yb^3+^ and 10% Er^3+^ ratio, balanced EM and BET effects, while a 1.5 nm inert shell provided optimal protection for the active core. Leveraging the photostable and background‐free properties of these optimized UCNPs, we developed UCNPs@WGA complexes to monitor axonal transport in DRG neurons. Using HMM‐Bayes analysis, we identified three distinct motion states: diffusion, anterograde transport, and retrograde transport. Statistical analysis revealed coordinated kinesin‐dynein interactions, with anterograde transport acting as an intermediary to activate retrograde transport. This finding provides mechanistic insights into motor protein coordination in DRG neurons. We further examined how inhibitors and stimulants modulate axonal transport. Inhibitors disrupted kinesin‐dynein balance, reducing transport velocity and increasing state transitions, whereas stimulants enhanced efficiency and promoted motor protein cooperation. These results highlight the utility of uSPT as a powerful tool for dissecting neuronal dynamics. Collectively, this work not only establishes UCNPs as versatile probes for biological imaging but also deepens our understanding of neuronal transport mechanisms, offering new avenues for studying intracellular dynamics and motor protein interplay.

## 4 Experimental Section

### Synthesis of 11 nm core UCNPs

UCNPs were synthesized using a solvent thermal method.^[^ ^39^ ^]^ A 100 mL three‐necked flask was charged with 10 mL of octadecene (ODE), 10 mL of OA, 1 mL RECl_3_·6H_2_O aqueous solution (1.0 mol L^−1^). The mixture was heated to 160 °C for ≈50 min under N_2_ flow to remove the water. After cooling down to 110 °C, 2.03 g NaOA and 0.34 g NH_4_F were added, followed by stirring for 30 min to dissolve the salts. The solution was then heated to 290 °C under argon and maintained for 50 min. After cooling to room temperature (RT), a cyclohexane/ethanol mixture was added, and the resultant solution was centrifuged at 16099 × g for 10 min. The precipitate was washed three times with ethanol/cyclohexane (3:1, v/v) and dispersed in 10 mL cyclohexane. Other core samples were synthesized analogously by adjusting the rare‐earth metal composition.

### Synthesis of 14 nm Core‐Shell UCNPs

A 100 mL three‐necked flask containing 8 mL of octadecene (ODE), 3 mL of OA and 0.3 mL YbCl_3_·6H_2_O aqueous solution (1 mmol mL^−1^) was heated to 160 °C under N_2_ flow to remove the water. After cooling to RT, 0.03 g NaOH and 0.0417 g NH_4_F (dissolved in 3.75 mL methanol) and 3 mL of 11 nm core UCNPs in cyclohexane were added. The mixture was stirred for 30 min at RT, heated to 120 °C for 30 min, then to 290 °C for 30 min under argon atmosphere. Post‐cooling, a cyclohexane/ethanol mixture was added, and the resultant solution was centrifuged at 16099 × g for 10 min. The products were collected and washed three times with ethanol/cyclohexane (1:1, v/v), and dispersed in 10 mL cyclohexane. Other core‐shell samples were prepared similarly with adjusted rare‐earth metals.

### Synthesis of 17 nm Core‐Shell‐Shell UCNPs

The core‐shell‐shell UCNPs were synthesized by depositing an inert shell onto core‐shell UCNPs. The procedure mirrored core‐shell synthesis, substituting the 11 nm core with core‐shell UCNPs and YbCl_3_·6H_2_O with LuCl_3_·6H_2_O. Other core‐shell‐shell variants were prepared analogously.

### Synthesis of Yb_0.9_Er_0.1_@Yb@Lu UCNPs with Varying Inert Shell Thickness

A shell precursor was prepared by mixing 0.03, 0.06, 0.10, 0.13, 0.22, 0.46 or 0.80 mL LuCl_3_·6H_2_O water solution (1 M) with 3 mL oleic acid and 8 mL 1‐octadecene in a 100 mL flask. The mixture was stirred under nitrogen atmosphere and heated to 160 °C for 40 min to remove the water. After cooling to RT, 3 mL of 14 nm core‐shell Yb_0.9_Er_0.1_@Yb@Lu in cyclohexane and a methanol solution containing NH_4_F/NaOH were added, stirred at RT for 30 min, heated to 120 °C (30 min), then to 290 °C (13, 20, 30, 35, 40, 50 or 75 min) under argon atmosphere. Purification followed the 14 nm core‐shell UCNP protocol described above. Final products Yb_0.9_Er_0.1_@Yb@Lu with different inert shell thicknesses (0.5, 1.0, 1.5, 2.1, 3.3, 5.4 or 6.8 nm) were stored in 10 mL cyclohexane.

### Synthesis of UCNPs@DSPE(‐NHS)

A chloroform solution containing 5 mg DSPE‐mPEG (2000) and 1 mg DSPE‐mPEG‐NHS was combined with 0.3 mL cyclohexane dissolved ≈17 nm Yb_0.9_Er_0.1_@Yb@Lu core‐shell‐shell UCNPs in a 25 mL round bottom flask. after 30 min ultrasonication at room temperature, chloroform was removed using rotary evaporation. The resulting film was then hydrated with 10 mL of deionized water, centrifuged at low speed (112 × g, 5 min) to remove aggregates, and purified via ultracentrifugation (16099 × g, 15 min) to remove excess lipids. The pellet was subsequently dispersed in 0.5 mL PBS.

### Synthesis of UCNPs@WGA

UCNPs@DSPE(‐NHS) in PBS (0.5 mL) was mixed with 0.5 mL WGA aqueous solution (1 mg mL^−1^) and stirred for 6 h at RT. The mixture was dialyzed, diluted with neurobasal medium, and used for cell incubation.

### DRG Neurons Culture^[^ ^78^ ^]^

Dorsal root ganglia (DRG) were dissected from fetal Sprague‐Dawley (SD) rats at gestation days 16‐18. Pregnant rats were euthanized by cervical dislocation, and fetal DRG neurons were isolated in pre‐cooled medium. The isolated DRG neurons were selected and digested with papain (2 mg mL^−1^) and DNase in DMEM/F12 at 37 °C (5% CO_2_, 40 min). Digestion was halted with fetal bovine serum (FBS), and tissue was gently triturated, filtered (150‐mesh), and centrifuged (1000 rpm, 5 min). The pellet was resuspended in serum‐containing medium with Penicillin‐Streptomycin and plated on poly‐L‐lysine‐coated glass‐bottom dishes. After 24–48 h, the initial culture medium was replaced with a complete culture medium composed of Neurobasal medium supplemented with 2% B‐27, 1% Penicillin‐Streptomycin, 10 µM Ara‐C, and 100 ng mL^−1^ nerve growth factor (NGF). Subsequently, the culture medium was replaced every 3 days.

### Incubation of Live DRG Neurons with UCNPs@WGA

DRG neurons in 20 mm diameter confocal dishes were incubated with UCNPs@WGA (≈15 nmol mL^−1^) for 4 h, washed thrice with neurobasal medium, and imaged using a wide‐field microscope system equipped with Nikon 100× NA 1.49 Oil objective and a 976 nm fiber laser (BL976‐PAG900, Thorlabs).

### Treatment of DRG Neurons with Inhibitor cD and Stimulant DA

After 5‐7 days in culture, neurons were treated with 30 µM ciliobrevin D or 30 µM dopamine (inhibitor) (stimulant) for 2 h. Post‐treatment, UCNPs@WGA were added for long‐term tracking.

### Stability and Cytocompatibility Assessment

To evaluate the colloidal stability of the nanoparticles under both physiological and harsh conditions, UCNPs@DSPE‐mPEG were incubated in aqueous solutions with pH values ranging from 2.09 to 9.94 at 37 °C for 24 h. For serum stability tests, particles were incubated in PBS with or without 10% FBS for 24 h at 37 °C. Samples were characterized before and after incubation by steady‐state upconversion emission spectroscopy and TEM to assess changes in optical properties and morphology. Cytocompatibility was evaluated using primary hippocampal neurons, which were chosen instead of dorsal root ganglion neurons because primary hippocampal cultures can be obtained in sufficient quantity with high reproducibility from embryonic day 18 (E18) Sprague–Dawley rats, thereby ensuring adequate sample size and statistical power for toxicity testing. Cells were seeded in 96‐well plates at a density of 1 × 10⁴ cells/well and allowed to mature for 7 days in vitro. UCNPs@DSPE‐mPEG at concentrations ranging from 20 to 200 nmol mL^−1^ were added to the cultures for 24 h. Cell viability was then quantified using the Cell Counting Kit‐8 (CCK‐8), and absorbance at 450 nm was measured using a microplate reader.

### Single‐Particle Imaging and HMM‐Bayes Analysis

Single‐particle optical characterization was performed in microscope system with Nikon 100 × NA 1.49 Oil objective and a 980 nm continuous‐wave laser. And the single particle luminescence was record by an EMCCD (iXon Ultra 897, Andor). Live‐cell single‐particle tracking time‐lapse image sequences were acquired at 20 frames per second (50 ms exposure per frame) over a 10‐min recording duration per field of view at a power density of 11.6 kW cm^−2^. Nanoparticle trajectories were extracted using the TrackIt MATLAB package. To ensure analysis robustness, only trajectories with a minimum length of 150 consecutive frames and an average signal‐to‐noise ratio > 2 were retained for further analysis. Tracks exhibiting defocusing, sudden large displacements, or interference from nearby fluorescent particles were manually reviewed and excluded. State classification of particle motion was conducted using the HMM‐Bayes MATLAB package.^[^ ^65^ ^]^

### Statistical Analysis

All statistical analyses were conducted using OriginPro and MATLAB. Prior to analysis, fluorescence intensity data and single‐particle trajectory parameters were visually inspected, and outliers were excluded. Unless otherwise specified, all data were presented as mean ± standard deviation (SD). Sample sizes (n) were indicated in the figure legends and refer to the number of individual nanoparticles or transport trajectories analyzed. For HMM‐Bayes analysis of particle trajectories, only those longer than 150 frames (acquired at 20 frames per second) and with signal‐to‐noise ratio > 2 were included to ensure reliable state classification. Transition rate constants and dwell times were extracted by exponential fitting of state transition statistics.

## Conflict of Interest

The authors declare no conflict of interest.

## Supporting information



Supporting Information

## Data Availability

The data that support the findings of this study are available from the corresponding author upon reasonable request.
