# Supplementary material for: Enhanced Single‐Particle Upconversion Imaging via Energy Migration Boosting
Source: Adv Sci (Weinh). 2025 Aug 27;12(43):e10624. doi: 10.1002/advs.202510624 (PMC12631817; doi:10.1002/advs.202510624)
Supplement: Supplementary file 1 — Supporting Information [file ADVS-12-e10624-s001.docx]

**Supplementary Information**

**Enhanced Single-Particle Upconversion Imaging via Energy Migration Boosting**

Yanxin Zhang, Rongrong Wen, Tianli Zhai, Wenrui Zhang, Fan Ding, Huan Ling, Yunxiang Zhang*, Qian Liu*

Y. Zhang, R. Wen, T. Zhai, W. Zhang, F. Ding, H. Ling, Prof. Y. Zhang, Prof. Q. Liu

Department of Chemistry and Shanghai Key Laboratory of Molecular Catalysis and Innovative Materials

Fudan University

Shanghai 200438, China

E-mail: zyx@fudan.edu.cn, qianliu@fudan.edu.cn

**1. Theoretical analysis^[1]^**

To further understand the energy transfer process, we analyzed the upconversion dynamics theoretically. Five processes were considered: three energy transfer upconversion (ETU) processes from Yb^3+^ to Er^3+^, cross-relaxion (CR) between the Er^3+^ ions, and back energy transfer (BET) from Er^3+^ to Yb^3+^. An energy transfer diagram is shown in Figure S1.


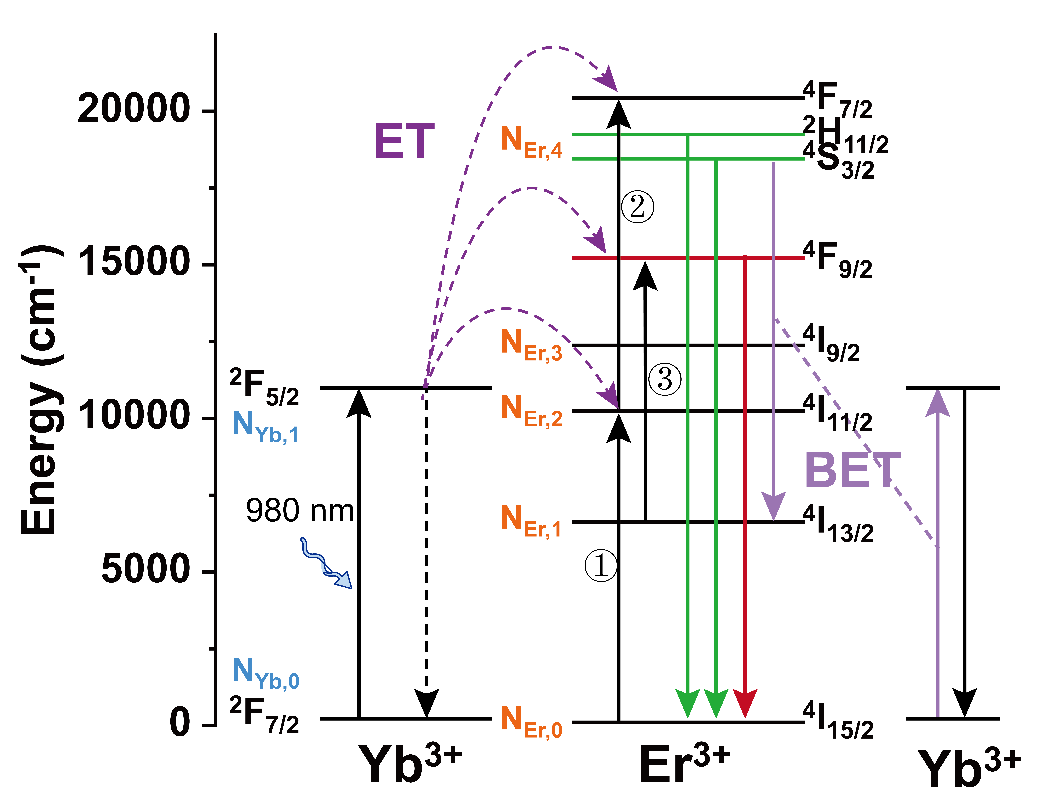


**Figure S1.** Schematic diagrams of energy transfer for theoretical analysis.

**ETU-1**: ^2^F_5/2_ (Yb^3+^) + ^4^I_15/2_ (Er^3+^) → ^2^F_7/2_ (Yb^3+^) + ^4^I_11/2_ (Er^3+^)

N_Yb,0_ + N_Er,0_ → N_Yb,1_ + N_Er,2_

**ETU-2**: ^2^F_5/2_ (Yb^3+^) + ^4^I_11/2_ (Er^3+^) → ^2^F_7/2_ (Yb^3+^) + ^4^F_7/2_ (Er^3+^)

N_Yb,0_ + N_Er,2_ → N_Yb,1_ + N_Er,4_

**ETU-3**: ^2^F_5/2_ (Yb^3+^) + ^4^I_13/2_ (Er^3+^) → ^2^F_7/2_ (Yb^3+^) + ^4^F_9/2_ (Er^3+^)

N_Yb,0_ + N_Er,1_ → N_Yb,1_ + N_Er,3_

**CR**: ^4^H_11/2_/^4^S_3/2_ (Er^3+^) + ^4^I_15/2_ (Er^3+^) → ^4^F_9/2_ (Er^3+^) + ^4^I_13/2_ (Er^3+^)

N_Er,4_ + N_Er,0_ → N _Er,3_ + N_Er,1_

**BET**: ^4^H_11/2_/^4^S_3/2_ (Er^3+^) + ^2^F_7/2_ (Yb^3+^) → ^4^I_13/2_ (Er^3+^) + ^2^F_5/2_ (Yb^3+^)

N_Yb,1_ + N_Er,4_ → N_Yb,0_ + N_Er,1_

Based on simplified process, we can be introduced the following steady-state rate equations:

$$\frac{dN_{Er,4}}{dt}=k_{2}N_{Er,2}N_{Yb,1}-R_{4}N_{Er,4}-W_{nr,4}N_{Er,4}-BN_{Er,4}N_{Yb,0}-CN_{Er,4}N_{Er,0}$$

$$\frac{dN_{Er,3}}{dt}=k_{3}N_{Er,1}N_{Yb,1}-R_{3}N_{Er,3}-W_{nr,3}N_{Er,3}$$

$$\frac{dN_{Er,2}}{dt}=k_{1}N_{Er,0}N_{Yb,1}-k_{2}N_{Er,2}N_{Yb,1}-W_{nr,2}N_{Er,2}$$

$$\frac{dN_{Er,1}}{dt}=W_{nr,2}N_{Er,2}+BN_{Er,4}N_{Yb,0}+CN_{Er,4}N_{Er,0}-k_{3}N_{Er,1}N_{Yb,1}-W_{nr,1}N_{Er,1}$$

$$\frac{dN_{Yb,1}}{dt}=\frac{P\sigma N_{Yb,0}}{hv}+BN_{Er,4}N_{Yb,0}-\left( k_{1}N_{Er,0}{+k_{2}N_{Er,2}+k}_{3}N_{Er,1} \right)N_{Yb,1}-RN_{Yb,1}$$

In this diagram,$N_{Er,i}\left( i=0,1,2,3,4 \right)$ are the population density of ^4^I_15/2_, ^4^I_13/2_, ^4^I_11/2_, ^4^F_9/2_, and ^4^H_11/2_/^4^S_3/2_ energy levels respectively of Er^3+^, R_i_ and W_nr,I_ are the corresponding radiative and non-radiative decay rates. $N_{Yb,i}\left( i=0,1 \right)$ are the population densities of ^4^F_7/2_ and ^4^F_5/2_ energy levels respectively of Yb^3+^, R is the radiative decay rate of the excited state. *k*_1_, *k*_2_ and *k*_3_ are the energy transfer rates of ETU-1, ETU-2 and ETU-3 respectively. C is the CR rate, and B is the back energy transfer rate to the Yb^3+^ ions. $\sigma$ is the absorption cross-section between energy level ^4^F_7/2_ and ^4^F_5/2_, P is the incident pump power.

In this Lu_0.9-x_Yb_x_Er_0.1_@Yb@Lu series, the Yb^3+^ ions concentration is more than 10-fold higher than the Er^3+^ ions concentration, Yb^3+^ ions also have much larger absorption cross-section compared to Er^3+^ at the 980 nm excitation wavelength. Thus, the absorption of NIR photons by Er^3+^ ions can be neglected, and the Er^3+^ ion content is fixed in the series, the CR can also be neglected.

At low excitation power and low sensitizers concentration, BET from the Yb^3+^ excitation state can be neglected. The ETU loss in ^4^I_13/2_, ^4^I_11/2_ levels is lower than the spontaneous decay rates. The population of these energy levels can be described by the following equations:

$$N_{Yb,1}=\frac{P\sigma N_{Yb,0}}{hv\left( R+k_{1}N_{Er,0}{+k_{2}N_{Er,2}+k}_{3}N_{Er,1} \right)} \propto P$$

$$N_{Er,1}=\frac{W_{nr,2}}{W_{nr,1}}N_{Er,2}+\frac{B}{W_{nr,1}}N_{Er,4}N_{Yb,0}$$

$$N_{Er,2}=\frac{k_{1}}{W_{nr,2}}N_{Er,0}N_{Yb,1}$$

$$N_{Er,4}=\frac{k_{2}}{\left( R_{4}+W_{nr,4}+BN_{Yb,0} \right)}N_{Er,2}N_{Yb,1}\propto P^{2}$$

$$N_{Er,3}=\frac{k_{3}}{\left( R_{3}+W_{nr,3} \right)}N_{Er,1}N_{Yb,1}=\frac{{k_{3}W}_{nr,2}}{{\left( R_{3}+W_{nr,3} \right)W}_{nr,1}}N_{Er,2}N_{Yb,1}+\frac{Bk_{3}}{\left( R_{3}+W_{nr,3} \right)W_{nr,1}}N_{Er,4}N_{Yb,0}N_{Yb,1}\propto aP^{2}+bP^{3}$$

Here, we observe that green emission exhibits two-photon dependency and the red emission also exhibits two-photon dependency when CR and BET is neglected (i.e. B=0 & C=0).

At high power and high Yb^3+^ doping, ETU loss in the ^4^I_13/2_, ^4^I_11/2_ levels become the main factor of de-population. The population of these energy levels can be described by following equations:

$$N_{Er,1}=\frac{W_{nr,2}}{k_{3}N_{Yb,1}}N_{Er,2}+\frac{B}{k_{3}N_{Yb,1}}N_{Er,4}N_{Yb,0}$$

$$N_{Er,2}=\frac{k_{1}}{k_{2}}N_{Er,0} \propto P^{0}$$

$$N_{Er,4}=\frac{k_{2}}{\left( R_{4}+W_{nr,4}+BN_{Yb,0} \right)}N_{Er,2}N_{Yb,1}\propto P$$

$$N_{Er,3}=\frac{k_{3}}{R_{3}}N_{Er,1}N_{Yb,1}=\frac{W_{nr,2}}{R_{3}N_{Yb,1}}N_{Er,2}N_{Yb,1}+\frac{B}{R_{3}N_{Yb,1}}N_{Er,4}N_{Yb,0}N_{Yb,1}\propto aP+bP^{2}$$

It is evident that the slope will decrease with increasing power, which is consistent with our experimental results. With increasing Yb^3+^ ions in the core, deeper energy transfer increases, except for interface energy transfer. The EBT and ETU-3 effects also increases, which benefits the ^4^F_9/2_ (Er^3+^) population and reduces the ^4^H_11/2_/^4^S_3/2_ (Er^3+^) population. Thus, the R/G ratio increases as well.

When considered CR in “alloy-core” architecture at low excitation and low sensitizer concentration, the solution becomes：

$$N_{Yb,1}=\frac{P\sigma N_{Yb,0}}{hv\left( R+k_{1}N_{Er,0}{+k_{2}N_{Er,2}+k}_{3}N_{Er,1} \right)} \propto P$$

$$N_{Er,1}=\frac{W_{nr,2}}{W_{nr,1}}N_{Er,2}+\frac{B+C}{W_{nr,1}}N_{Er,4}N_{Yb,0}$$

$$N_{Er,2}=\frac{k_{1}}{W_{nr,2}}N_{Er,0}N_{Yb,1}$$

$$N_{Er,4}=\frac{k_{2}}{\left( R_{4}+W_{nr,4}+BN_{Yb,0}+CN_{Yb,0} \right)}N_{Er,2}N_{Yb,1}\propto P^{2}$$

$$N_{Er,3}=\frac{k_{3}}{\left( R_{3}+W_{nr,3} \right)}N_{Er,1}N_{Yb,1}=\frac{{k_{3}W}_{nr,2}}{{\left( R_{3}+W_{nr,3} \right)W}_{nr,1}}N_{Er,2}N_{Yb,1}+\frac{(B+C)k_{3}}{\left( R_{3}+W_{nr,3} \right)W_{nr,1}}N_{Er,4}N_{Yb,0}N_{Yb,1}\propto aP^{2}+bP^{3}$$

At high power and high Yb doping, the corresponding solution is:

$$N_{Er,1}=\frac{W_{nr,2}}{k_{3}N_{Yb,1}}N_{Er,2}+\frac{B+C}{k_{3}N_{Yb,1}}N_{Er,4}N_{Yb,0}$$

$$N_{Er,2}=\frac{k_{1}}{k_{2}}N_{Er,0} \propto P^{0}$$

$$N_{Er,4}=\frac{k_{2}}{\left( R_{4}+W_{nr,4}+BN_{Yb,0}+CN_{Yb,0} \right)}N_{Er,2}N_{Yb,1}\propto P$$

$$N_{Er,3}=\frac{k_{3}}{R_{3}}N_{Er,1}N_{Yb,1}=\frac{W_{nr,2}}{R_{3}N_{Yb,1}}N_{Er,2}N_{Yb,1}+\frac{B+C}{R_{3}N_{Yb,1}}+cN_{Er,4}N_{Yb,0}N_{Yb,1}\propto aP+bP^{2}$$

Obviously, B+C can be considered as a single parameter to discuss its impact on the population density. In this configuration, the relationship between B and C is inversely related. So there will be a minimum value to achieve the maximum population density. Through experimental verification, the most effective way to achieve upconversion luminescence is using Yb_0.9_Er_0.1_@Yb@Lu as the ultimate formula.

**Supplementary Note: Sources of variability in single-particle brightness measurements**

The standard deviation observed in single-particle brightness stems from a combination of intrinsic material heterogeneity and measurement-related fluctuations.

1. Intrinsic material heterogeneity

Particle size and morphology heterogeneity: Although our synthetic protocol yields sub-20 nm nanoparticles with a narrow size distribution (as shown in TEM), slight variations in diameter (±1-2 nm) can significantly influence the effective emitter volume and energy migration distance, which directly affects brightness.

Dopant distribution inhomogeneity: The distribution of Yb^3+^ and Er^3+^ ions within the lattice may not be perfectly uniform due to kinetic factors during shell growth. These local deviations can affect energy transfer dynamics, contributing to particle-to-particle brightness variation.

Surface passivation effectiveness: The effectiveness of the final shell growth may vary slightly from particle to particle, leading to differences in surface quenching suppression. Variations in the thickness uniformity or crystallinity of the inert outer shell directly influence brightness by affecting surface-related non-radiative losses.
2) Experimental Measurement Factors

Photon counting statistics and detection noise: At low excitation powers, the signal-to-noise ratio is limited and single-photon detection introduces Poisson noise and system-level fluctuations, contributing to measurement uncertainty.

Optical alignment and z-position drift: Despite employing precise tracking protocols, minor differences in particle focus due to axial drift during long exposure times can affect detected brightness.

Despite these unavoidable factors, we want to emphasize the overall brightness enhancement trend is statistically significant and reproducible, as confirmed across three independent batches. These variations reflect the real heterogeneity of nanomaterials under biologically relevant conditions and underscore the importance of performing single-particle level measurements to capture the true distribution of photophysical properties.


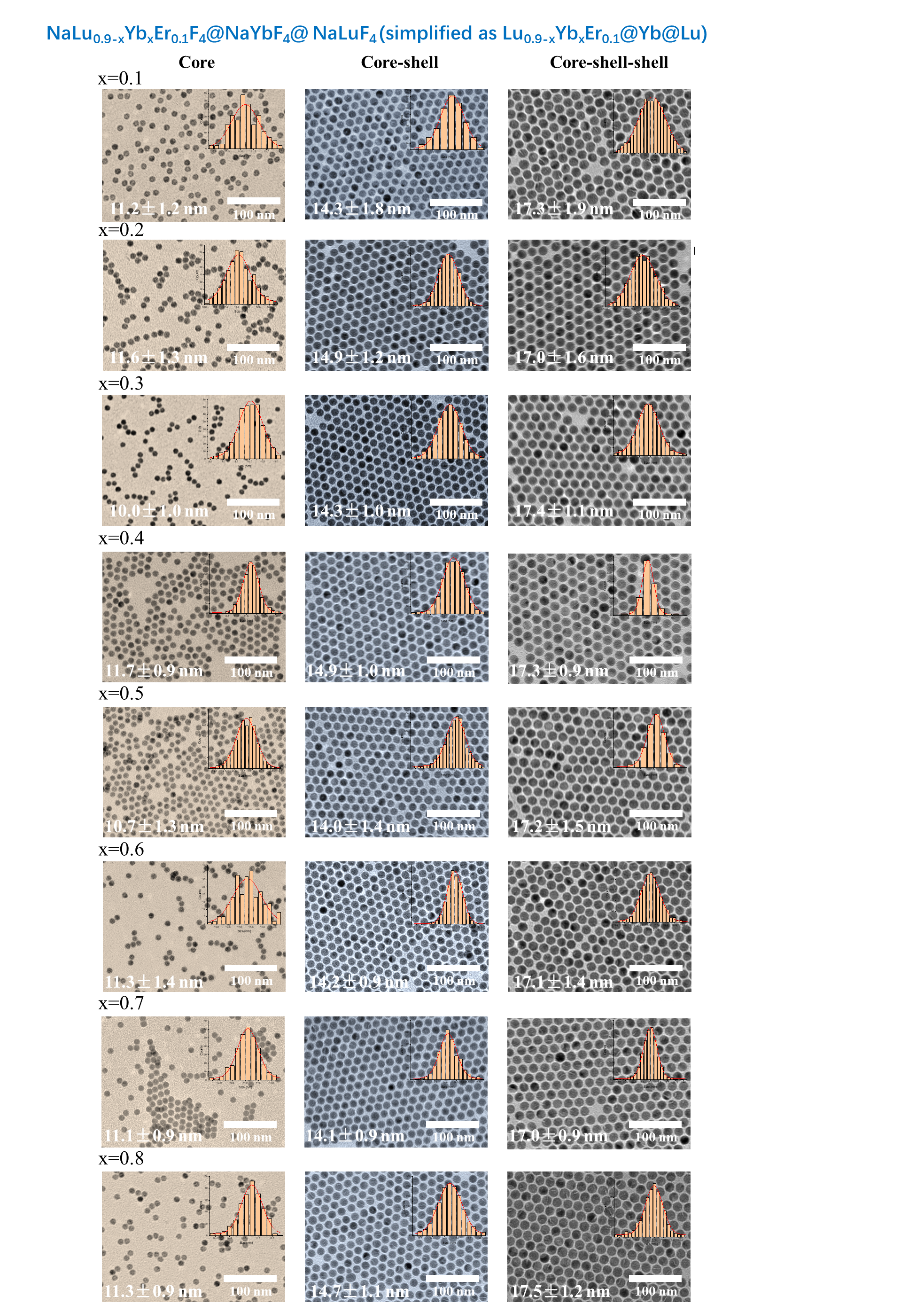
**2. Characterization of UCNPs**

**Figure S2.** TEM images of Lu_0.9-x_Yb_x_Er_0.1_@Yb@Lu UCNPs. TEM images of core (left), core-interior shell (center) and final core-interior shell-inert shell UCNPs (right) at different doping concentration. Each panel includes a size distribution histogram with a Gaussian fit. The mean size (from the Gaussian fit) and standard deviation are indicated in the lower-left corner of each image.


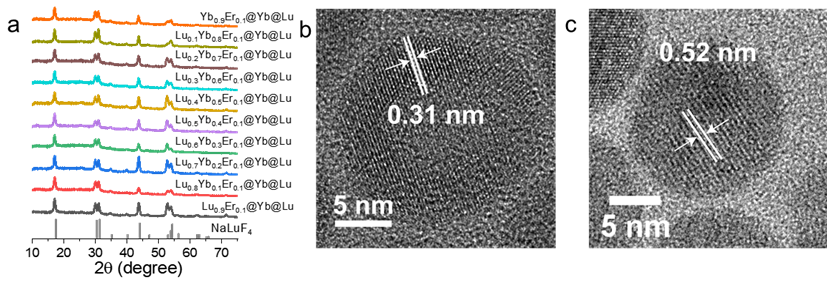


**Figure S3.** Structural characterization of Lu_0.9-x_Yb_x_Er_0.1_@Yb@Lu UCNPs. (a) XRD patterns of the core- shell- shell samples, compared with the reference spectrum of hexagonal β-NaLuF_4_ (JCPDS #27-0726). (b-c) HR-TEM image of Yb_0.9_Er_0.1_@Yb@Lu, showing a lattice spacing of 0.31 nm and 0.52 nm, corresponding to the (100) and (101) planes. Scale bar, 5 nm.


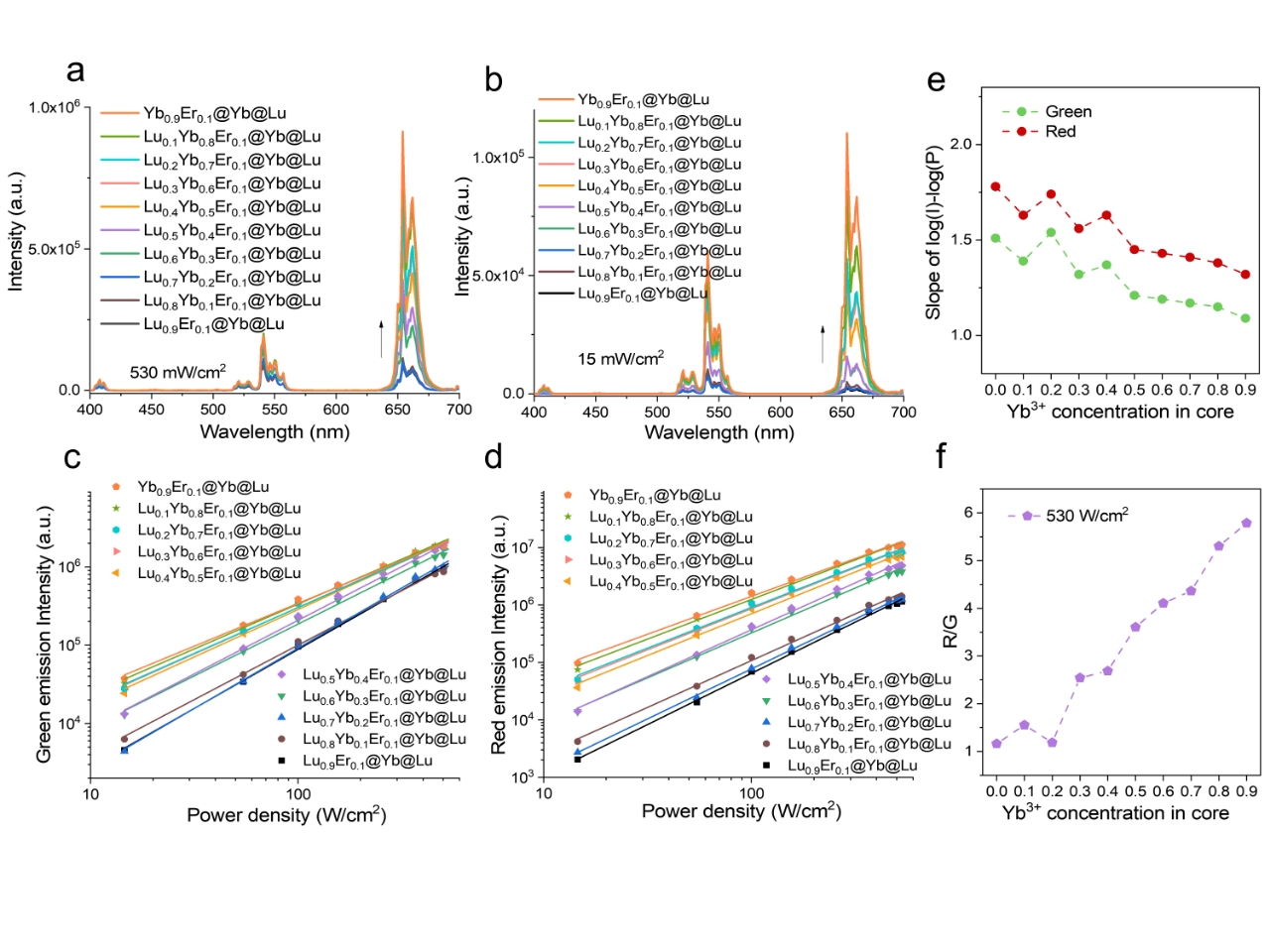


**Figure S4.** Ensemble UCL characterization of Lu_0.9-x_Yb_x_Er_0.1_@Yb@Lu UCNPs. (a) UCL spectra of ensemble UCNPs in cyclohexane solution under 530 W cm^-2^ and (b)15 W cm^-2^ 980 nm laser excitation. (c-d) Power dependence of ensemble UCL red emission at 654 nm and green emission at 541 nm, with linear regression used to determine (e) the corresponding slopes. (f) The R/G ratio under 530 W cm^-2^ 980 nm laser irradiation.

**
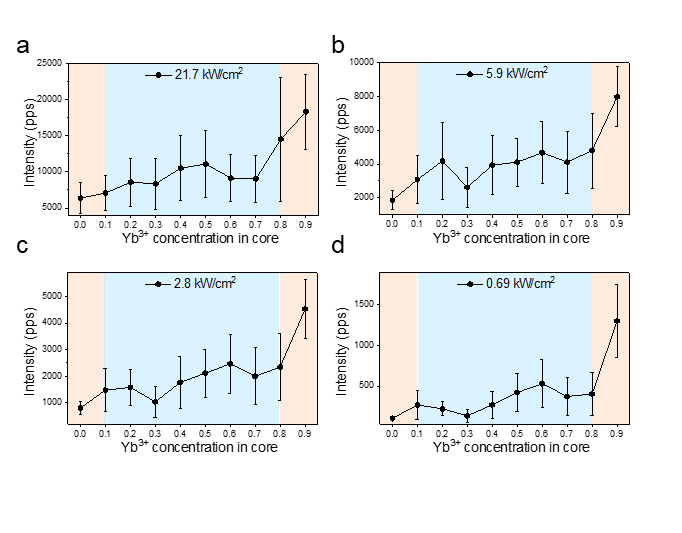
**

**Figure S5.** Single-particle upconversion luminescence intensities of Lu_0.9-x_Yb_x_Er0.1@Yb@Lu UCNPs at varying excitation power densities. single-particle upconversion luminescence intensities of Lu_0.9-x_Yb_x_Er0.1@Yb@Lu UCNPs at excitation power densities of 21.7, 5.9, 2.8, and 0.69 kW cm^-2^. The results were presented as means ± standard deviation (two independent experiments, more than five field of views of wide-field images were acquired for each experiments; "n" represents the number of single nanoparticles, Lu_0.9_Er_0.1_@Yb@Lu: n = 313, Lu_0.8_Yb_0.1_Er_0.1_@Yb@Lu: n = 527, Lu_0.7_Yb_0.2_Er_0.1_@Yb@Lu: n = 415, Lu_0.6_Yb_0.3_Er_0.1_@Yb@Lu: n = 226, Lu_0.5_Yb_0.4_Er_0.1_@Yb@Lu: n = 355, Lu_0.4_Yb_0.5_Er_0.1_@Yb@Lu: n = 238, Lu_0.3_Yb_0.6_Er_0.1_@Yb@Lu: n = 713, Lu_0.2_Yb_0.7_Er_0.1_@Yb@Lu: n = 625, Lu_0.1_Yb_0.8_Er_0.1_@Yb@Lu: n = 426, Yb_0.9_Er_0.1_@Yb@Lu: n = 686), "pps" means photons per second, and "pps px^-1^" means photons per second per pixel.

**
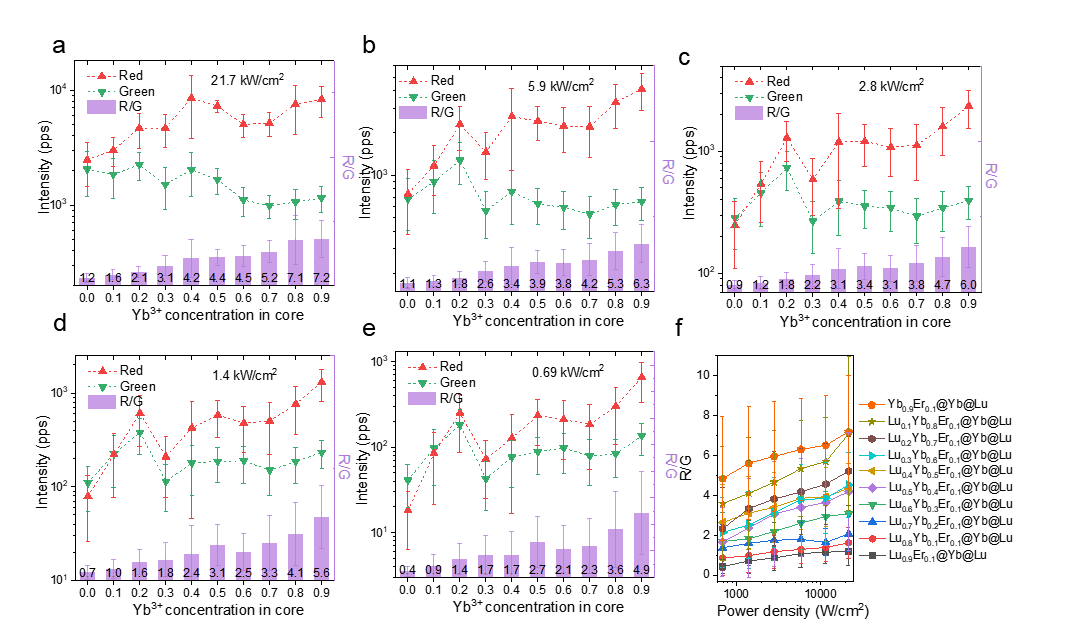
Figure S6.** Spectral analysis of single-particle upconversion luminescence of Lu_0.9-x_Yb_x_Er_0.1_@Yb@Lu UCNPs. (a-e) Green and red emission of single UCNPs, measured using ET535/70m (green) and ET645/75m (red) bandpass filters, the corresponding R/G ratio at excitation power densities of 21.7, 5.9, 2.8, 1.4- and 0.69-kW cm^-2^. (f) The R/G ratio of Lu_0.9-x_Yb_x_Er_0.1_@Yb@Lu UCNPs at various power densities.


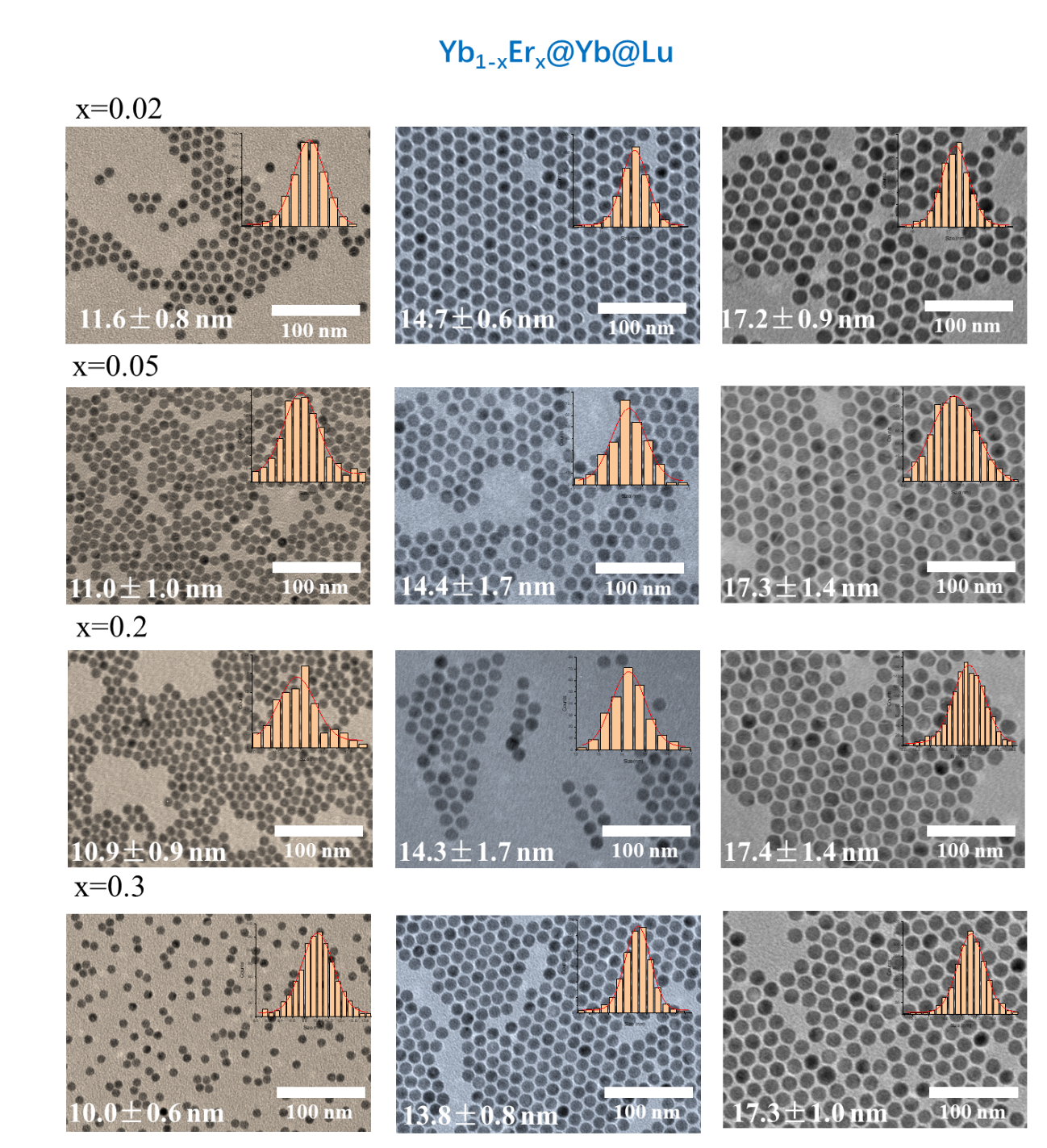


**Figure S7.** TEM images of Yb_1-x_Er_x_@Yb@Lu UCNPs. TEM images showing the core (left), core-interior shell (center) and final core-interior shell-inert shell UCNPs (right) at different doping concentration. Each panel includes a size distribution histogram with a Gaussian fit. The mean size (from the Gaussian fit) and standard deviation are indicated in the lower-left corner of each image.


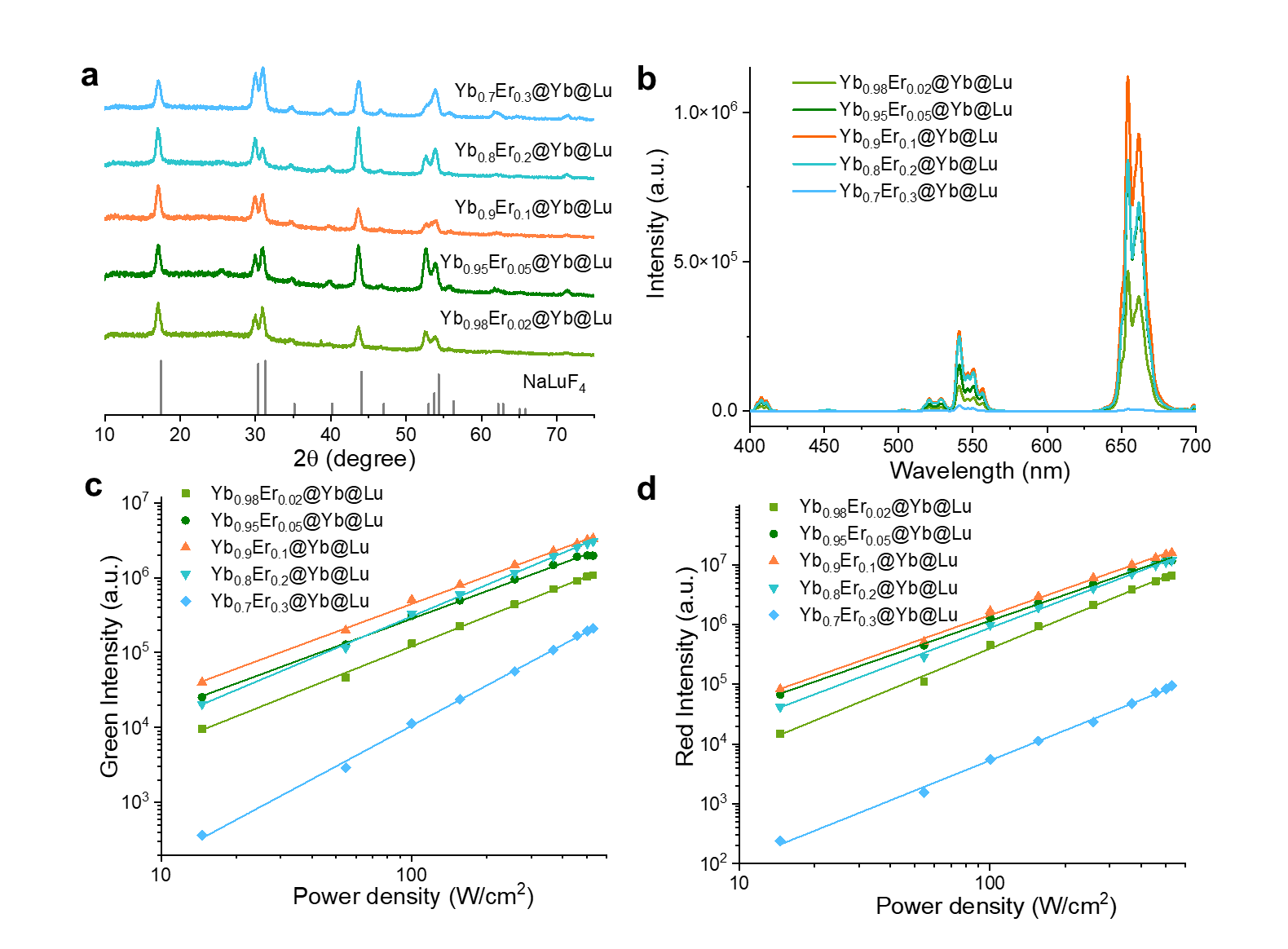


**Figure S8.** Structural and spectroscopic characterization of Yb_1-x_Er_x_@Yb@Lu UCNPs. (a) XRD pattern of the synthesized samples, compared with the reference spectrum for hexagonal β-NaLuF_4_ (JCPDS #27-0726). (b) Ensemble UCL spectra of UCNPs in cyclohexane solution under 530 $W \mathrm{cm}^{-2}$980 nm laser excitation. (c-d) Power dependence of green emission at 541 nm and red emission at 654 nm.


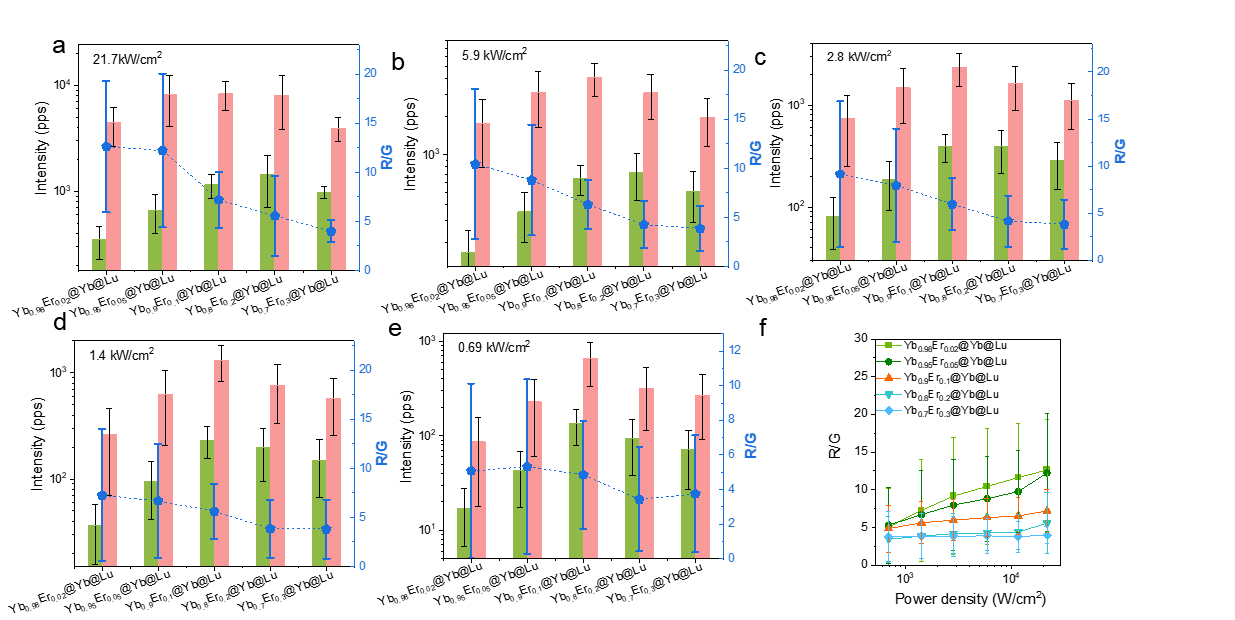


**Figure S9.** Spectral analysis of single-particle upconversion luminescence for Yb_1-x_Er_x_@Yb@Lu UCNPs. (a-e) Green and red emission intensities of single UCNPs, measured using ET535/70m (green) and ET645/75m (red) bandpass filters, and corresponding R/G ratio at power densities of 21.7, 5.9, 2.8, 1.4, and 0.69 kW cm^-2^. (f) R/G ratio of Yb_1-x_Er_x_@Yb@Lu at various power densities. The results were presented as means ± standard deviation (two independent experiments, more than five field of views wide-field images were acquired for each experiment; Yb_0.98_Er_0.02_@Yb@Lu：n = 615, Yb_0.95_Er_0.05_@Yb@Lu：n = 508, Yb_0.9_Er_0.1_@Yb@Lu：n = 958, Yb_0.8_Er_0.2_@Yb@Lu：n = 381, Yb_0.7_Er_0.3_@Yb@Lu：n = 270).


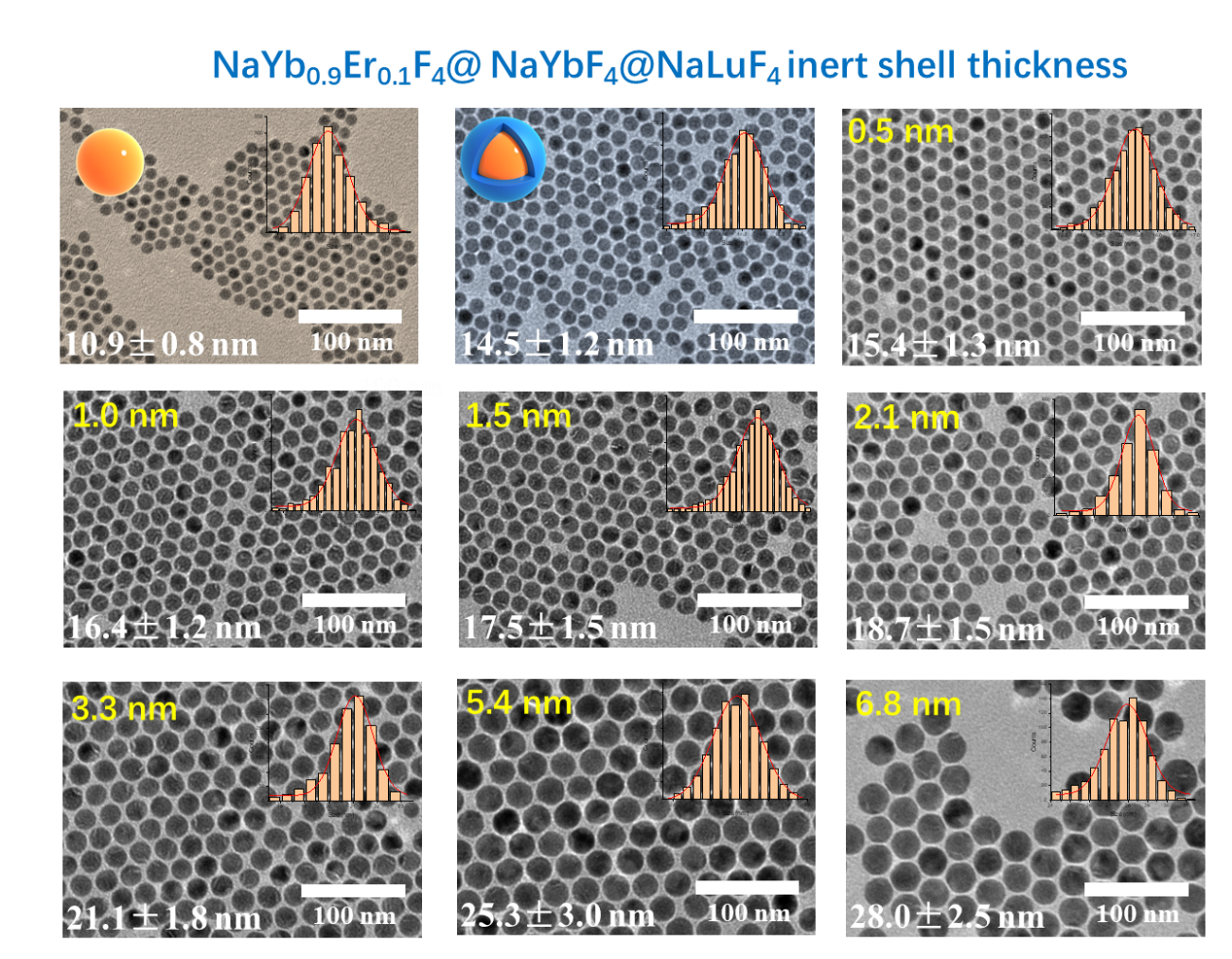


**Figure S10.** TEM images of Yb_0.9_Er_0.1_@Yb@Lu with varying inert shell thickness. Each panel includes a size distribution histogram with a Gaussian fit. The mean size (from the Gaussian fit) and standard deviation are indicated in the lower left corner of each panel. The samples presented here is the same as that presented in Figure 4a.


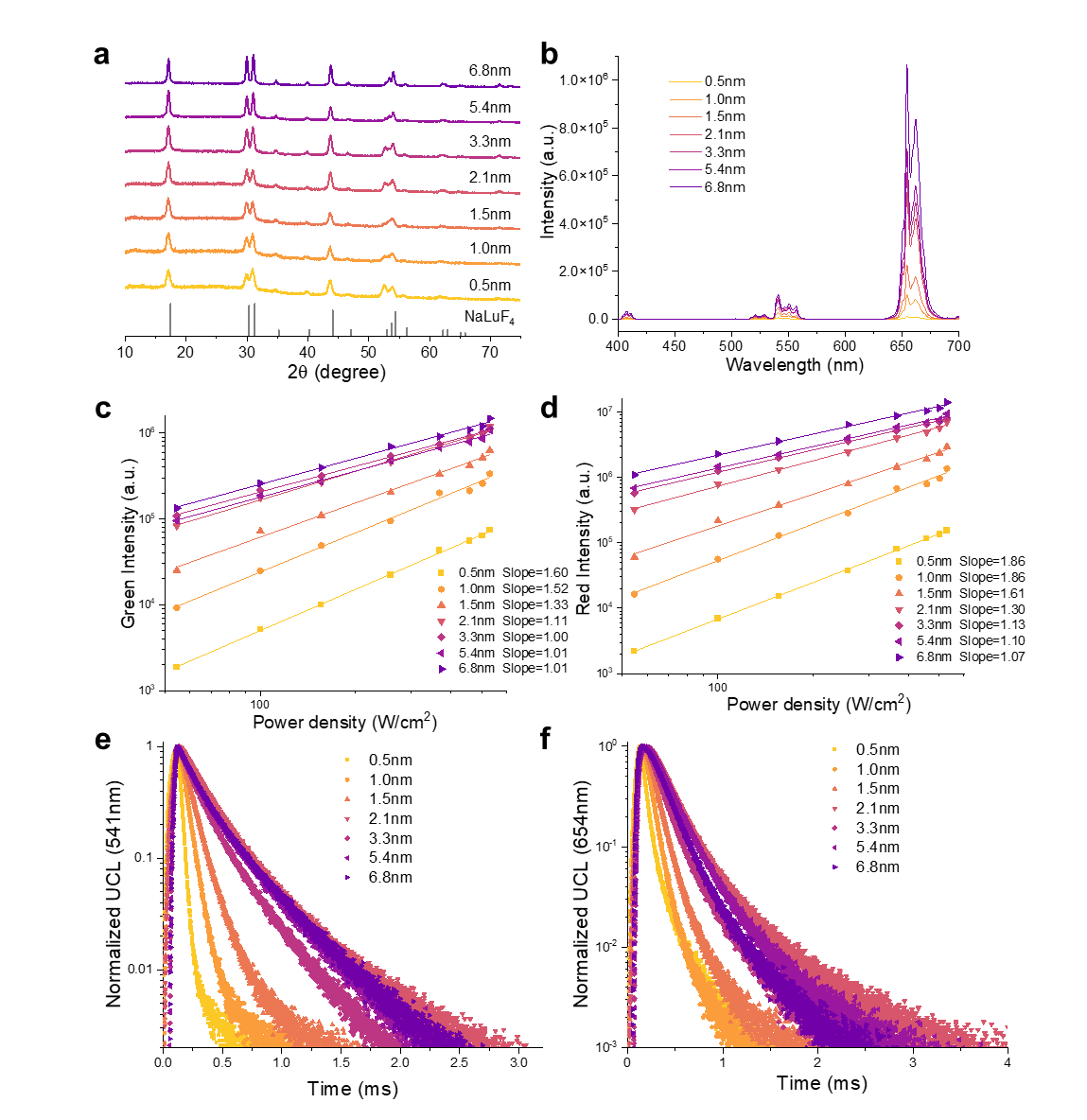


**Figure S11.** Structural and spectroscopic characterization of Yb_0.9_Er_0.1_@Yb@Lu with Varying Inert Shell Thickness. (a) XRD pattern of the synthesized samples, compared with the reference spectrum of hexagonal β-NaLuF_4_ (JCPDS #27-0726). (b) Ensemble UCL spectra of UCNPs in cyclohexane solution under 530 W cm^-2^ 980 nm laser excitation. (c-d) Power dependence of green (541 nm) and red (654 nm) emission intensities. Luminescence decay curves of UCNPs with different inert shell thickness, excited by a 980 nm pulsed laser and recorded at (e)541 nm; (f) 654 nm emission.


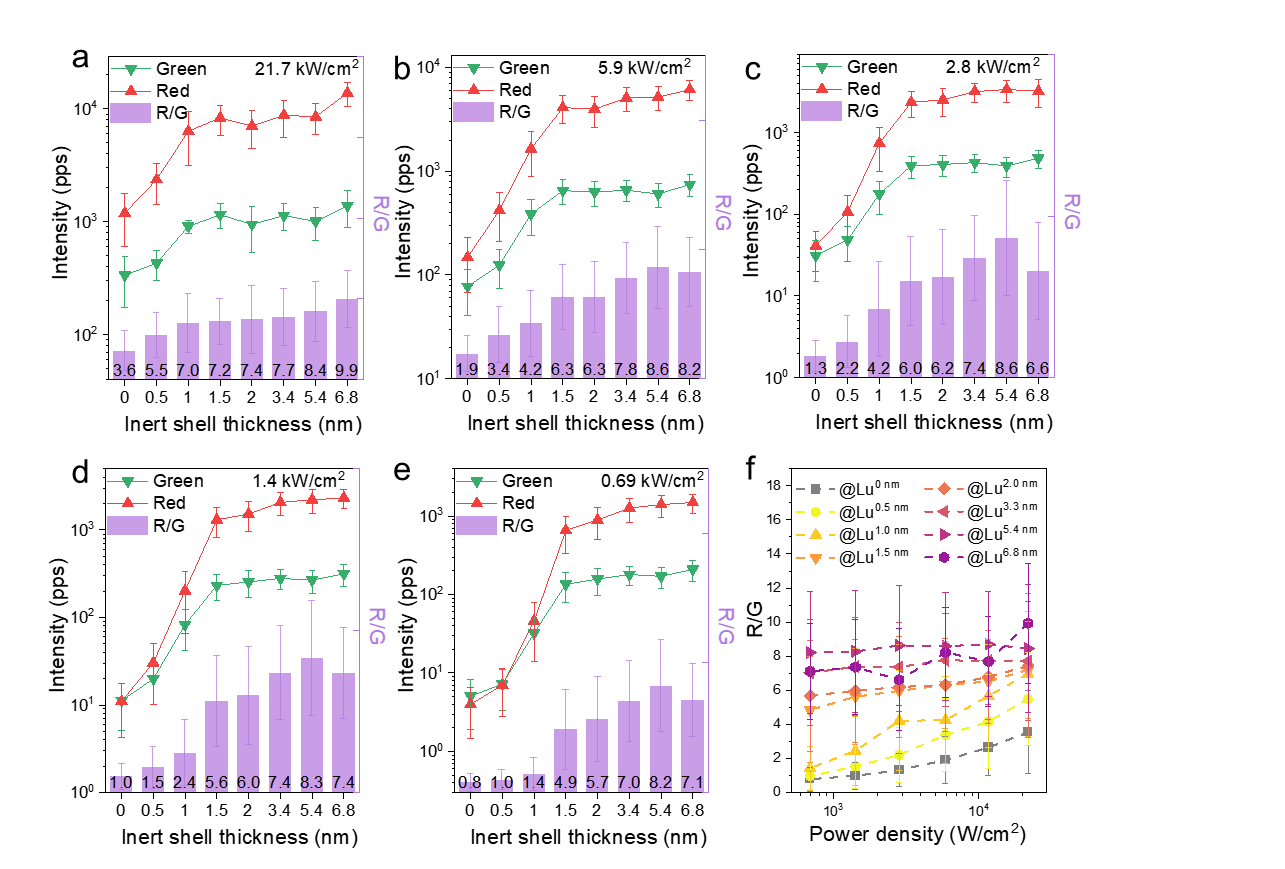


**Figure S12.** Spectral analysis of single-particle upconversion luminescence for Yb_0.9_Er_0.1_@Yb@Lu UCNPs with varying inert shell thickness (a-e) Green and red emission intensities of single UCNPs, measured using ET535/70m (green) and ET645/75m (red) bandpass filters, and the corresponding R/G ratio at power densities of 21.7, 5.9, 2.8, 1.4- and 0.69-kW cm^-2^. (f) R/G ratio of Yb_0.9_Er_0.1_@Yb@Lu with different inert shell thickness at various power densities. The results were presented as means ± standard deviation (two independent experiments, more than five field of views wide-field images were acquired for each experiment; 0 nm：n = 459, 0.5 nm：n = 258, 1.0 nm：n = 260, 1.5 nm：n = 460, 2.0 nm：n = 550, 3.3 nm：n = 656, 5.4 nm：n = 523, 0 nm：n = 213).

**Figure S13.** Upconversion luminescence spectra of UCNPs@DSPE-PEG incubation in aqueous solutions of different pH values (2.09, 4.01, 7.00, 9.21 and 9.94).


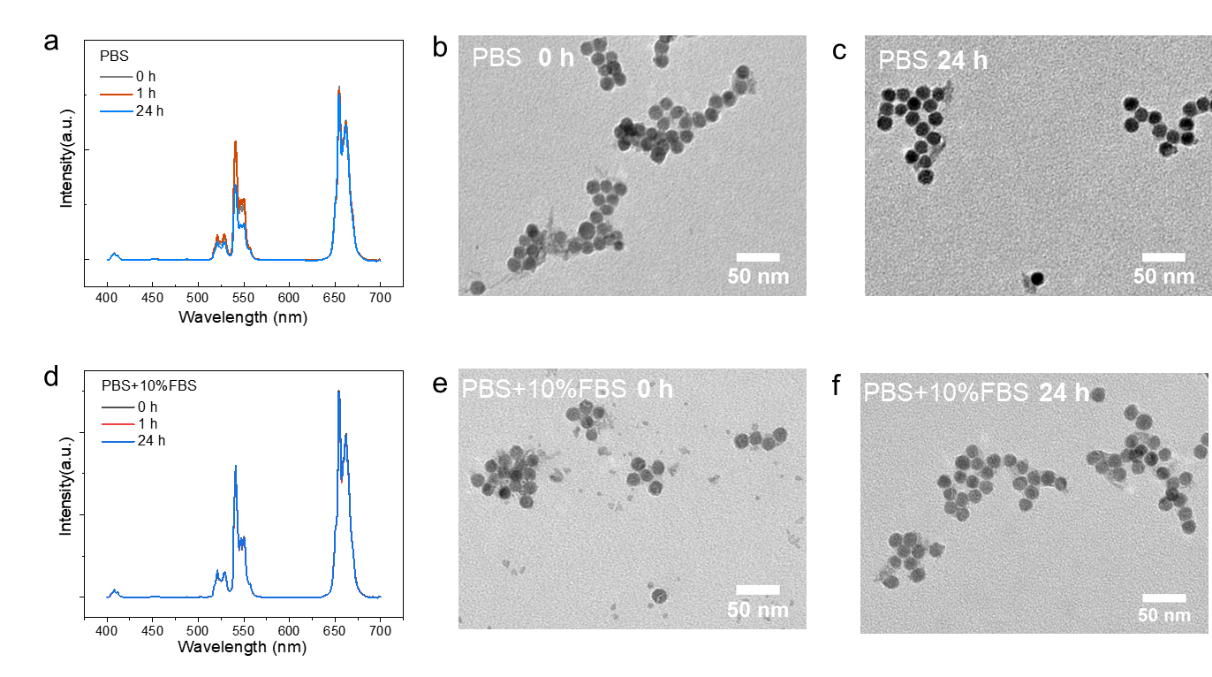


**Figure S14.** (a) UCL spectra of UCNPs@DSPE-PEG in serum-free PBS after 0 h, 1 h and 24 h. (b-c) Representative TEM images of UCNPs@DSPE-PEG before and after 24 h incubation in PBS. (d) UCL spectra of UCNPs@DSPE-PEG in 10% FBS-containing PBS after 0 h, 1 h and 24 h. (e-f) Representative TEM images of UCNPs@DSPE-PEG before and after 24 h incubation in PBS+10% FBS.


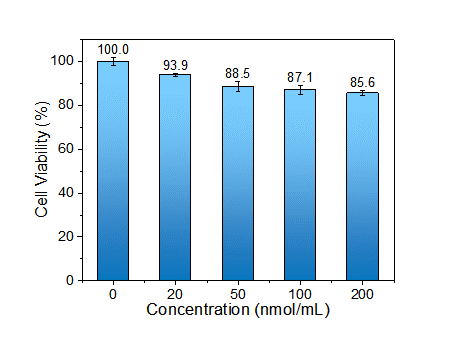


**Figure S15.** CCK-8 assay of neurons after 24 h exposure to different concentrations (20, 50, 100, 200 nmol/mL) of PEGylated UCNPs@DSPE-PEG.

**3. Axonal transport tracking**


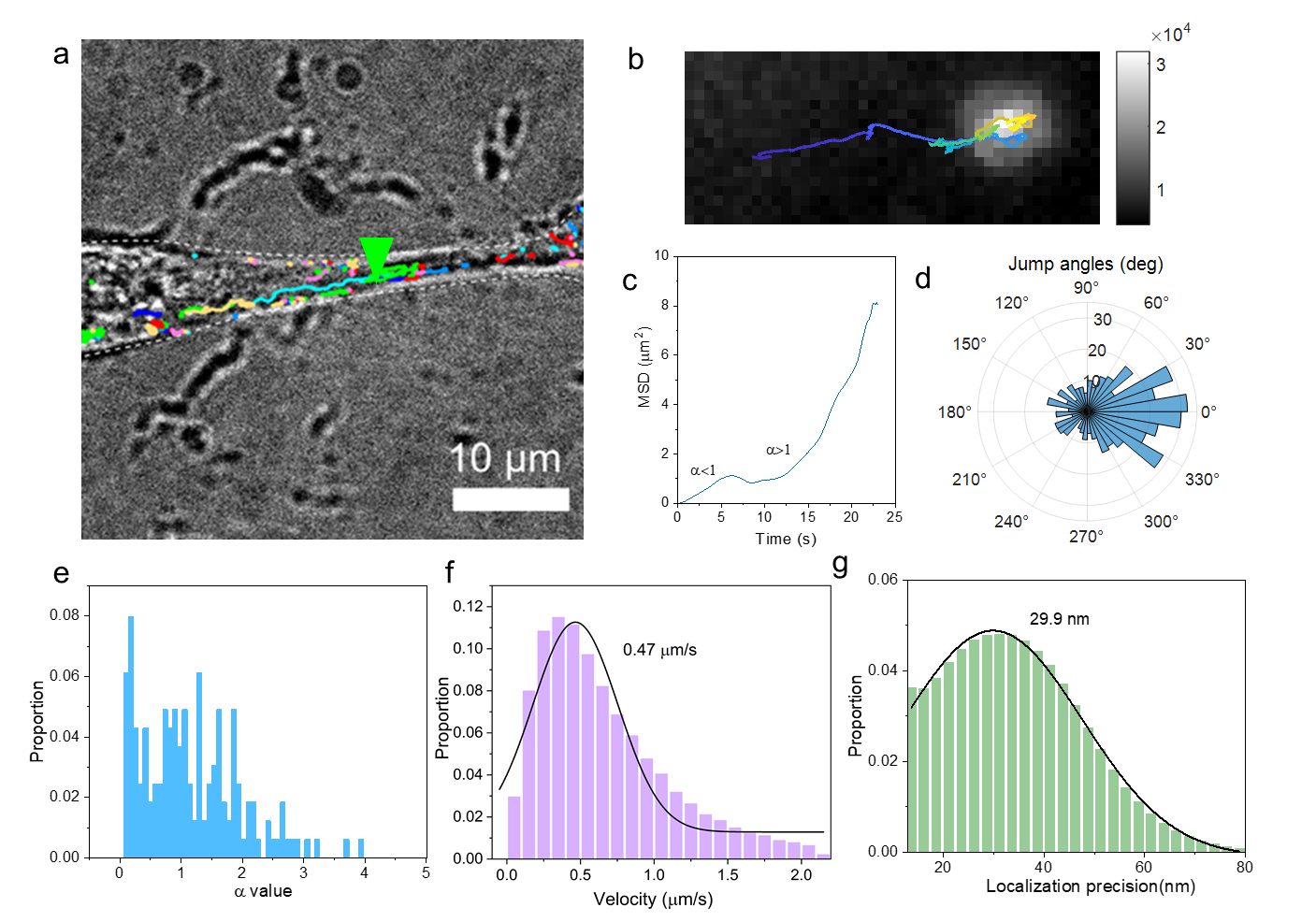


**Figure S16.** Analysis of UCNPs@WGA dynamics in neurons. (a) Bright field image of a neuron overlayed with all trajectories of randomly moving UCNPs@WGA. The green arrow indicates the location of the trajectory shown in (b). (b) Representative trajectory analysis of axon transport. (c) MSD curves and (d) jump angle distribution for the trajectory shown in (b). Distribution of (e) $\alpha$value, (f) velocity and (g) localization precision across all trajectories.

**Figure S17.** Effect of stimulants and inhibitors on UCNPs@WGA dynamics in neurons. Bright field images of neurons showing all trajectories of randomly moving UCNPs@WGA under the influence of (a) stimulants and (b) inhibitors.


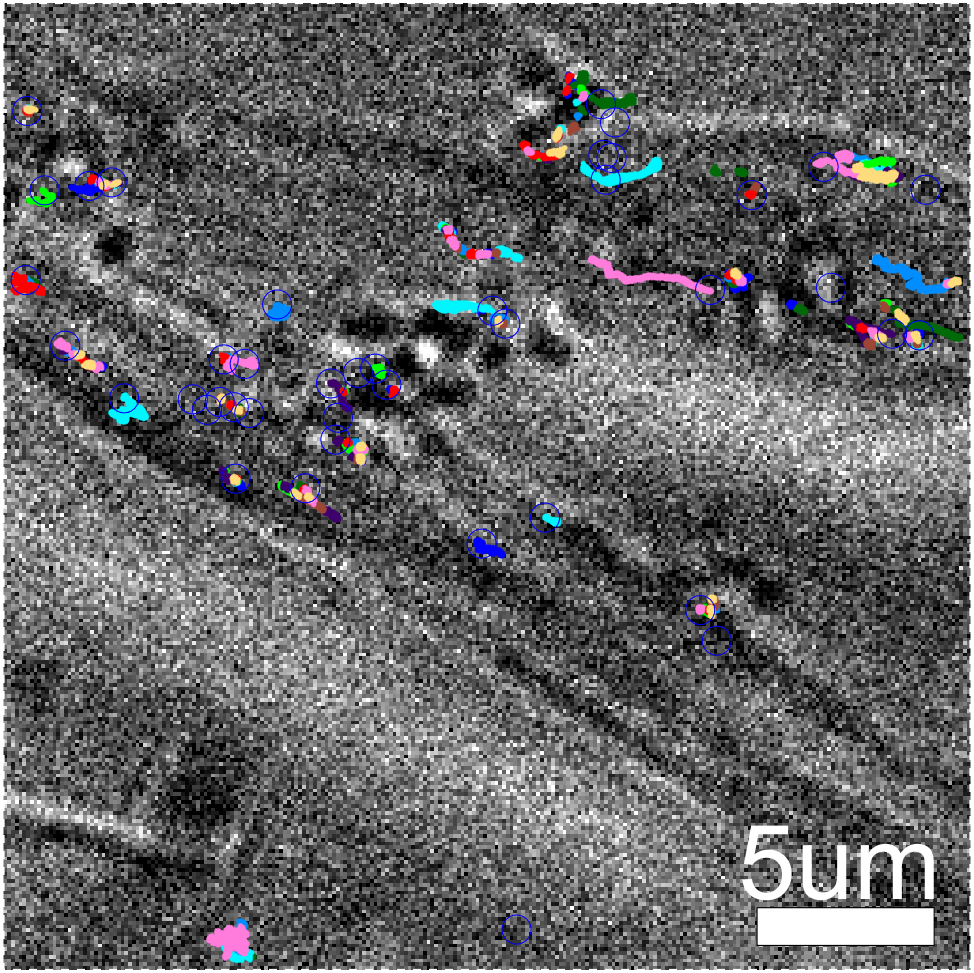


A


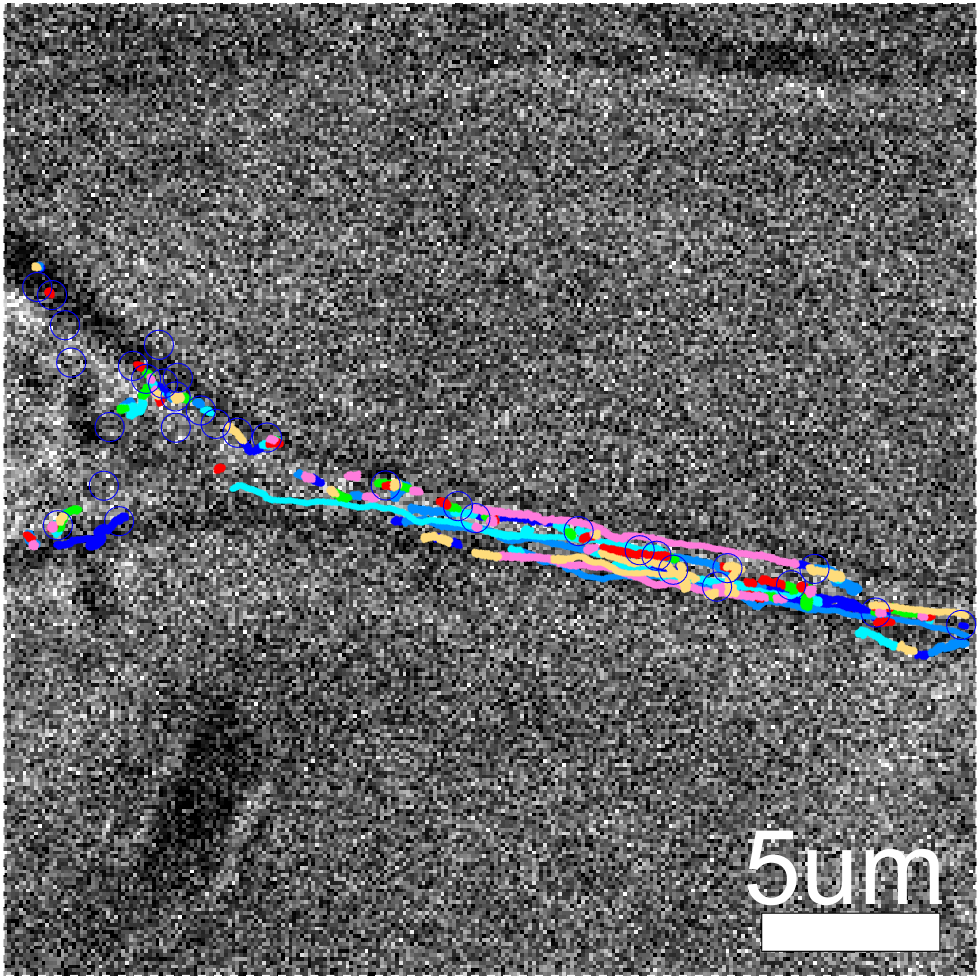


a

b

*
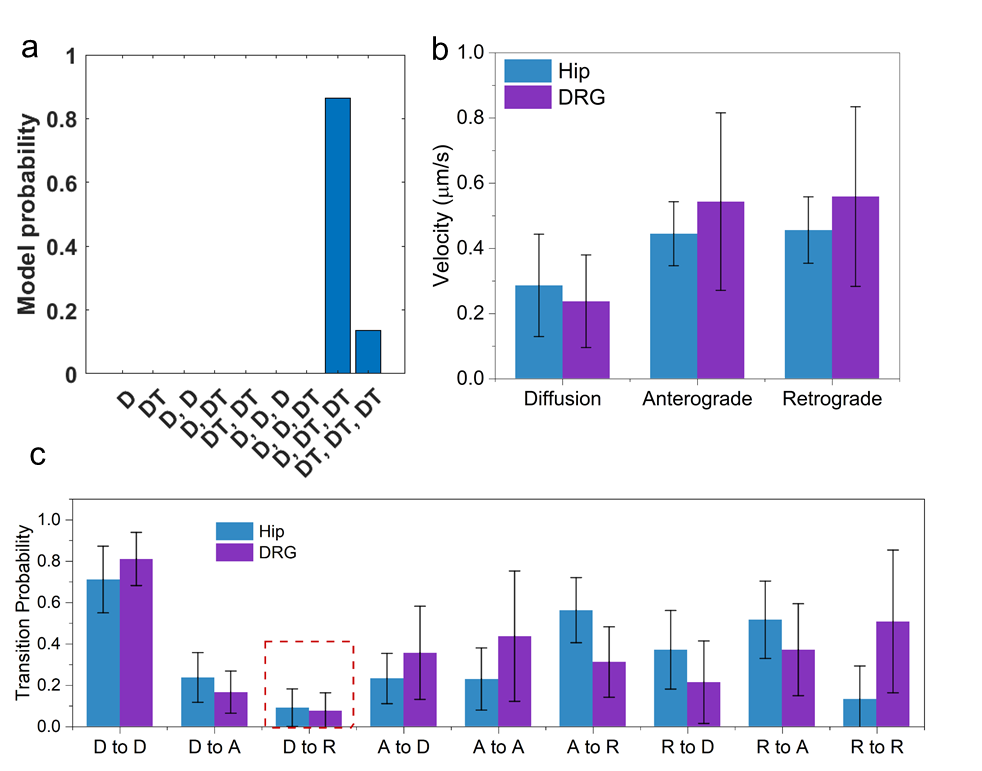
*

**Figure S18.** Upconversion Single-Particle Tracking of Axonal Transport in Hip Neurons. (a) Model probabilities for all motion models evaluated in the HMM-Bayes analysis. (b) Velocities distributions for the three HMM-Bayes states derived from all axonal transport trajectories. (c) transition probabilities of the three states.

**References**

[1] a)P. P. Sukul, K. Kumar, H. Swart, *Dalton Trans.* **2022**, *51*, 2827; b)J. Xu, A. Gulzar, P. Yang, H. Bi, D. Yang, S. Gai, F. He, J. Lin, B. Xing, D. Jin, *Coord. Chem. Rev.* **2019**, *381*, 104.
